# Profiles of medication literacy and associated factors among young and middle-aged hypertensive patients in China: A latent profile analysis

**DOI:** 10.1371/journal.pone.0352857

**Published:** 2026-07-22

**Authors:** Rui Wang, Chongbin Liu, Yu Wang, Xiaopu Shi, Siwen Ma, Gaokai Li, Gang Wang

**Affiliations:** 1 School of Life and Health, Huzhou College, Huzhou, Zhejiang, China; 2 School of Medicine, Huzhou University, Huzhou, Zhejiang, China; 3 Zhejiang Xinhua Hospital, Hangzhou, Zhejiang, China; Ningbo University, CHINA

## Abstract

Improving medication literacy among individuals with hypertension has emerged as a significant public health issue. However, the heterogeneity of medication literacy and its association factors among young and middle-aged patients with hypertension has not yet been fully characterized. The current study aimed to identify distinct profiles of medication literacy in these population and to examine their associated factors, with the goal of informing targeted clinical interventions. A cross-sectional study was conducted with 544 consecutively recruited these hypertensive patients from two tertiary hospitals in China between July 15 and November 15, 2024. Researchers administered five validated instruments: a Demographic and Disease-Related Characteristics Questionnaire, the Revised Chinese Medication Literacy Scale for Hypertensive Patients (C-MLSHP-R), the Perceived Social Support Scale (PSSS), the General Self-Efficacy Scale (GSES), and the Brief Illness Perception Questionnaire (BIPQ). Latent profile analysis revealed three distinct profiles of medication literacy: low (35.3%), moderate (50.2%), and high (14.5%). Multivariate logistic regression further identified education level, per capita monthly household income, social support, self-efficacy, and illness perception as factors significantly associated with medication literacy (all *p <* 0.05). More specifically, lower education and lower income were associated with membership in the low literacy profile, while higher social support, higher self-efficacy, and more positive illness perception characterized the high literacy group. These results suggest preliminary stratified interventions approaches, including foundational education for patients with low-literacy patients, behavioral reinforcement for those with moderate literacy, and enhanced psychosocial support for socioeconomically disadvantaged groups. However, causal interpretations are precluded by the cross-sectional design, longitudinal studies are needed to validate these findings.

## 1. Introduction

Currently, China is grappling with the escalating burden of hypertension. A recent study indicated 59% (aged 55–62) of women and 49% (aged 46–52) of men diagnosed with hypertension in 2019 [1]. An increasing prevalence of hypertension persists among young and middle-aged people, suggesting an increased risk of cardiovascular complications. What’s more, the focus on young and middle-aged adults is particularly warranted for several reasons. First, this age group represents a critical window for early intervention, as cardiovascular risk factors established during this period significantly influence later-life outcomes. Second, young and middle-aged hypertensive patients face unique challenges including work-related stress, family responsibilities in China. Third, these hypertensive patients demonstrate particular vulnerability due to three critical factors: heightened health risk perception deficits compared to older adults, inadequate self-management behaviors, and persistent misconceptions equating asymptomatic presentation with therapeutic non-necessity [2,3]. Hypertension prevalence in China has risen, while the awareness, treatment and control rates for hypertension remain relatively low [4]. Therefore, effective management of hypertension among young and middle-aged people is essential to prevent deterioration and complications, making it a primary concern in public health.

Medication literacy is an individual’s ability to acquire, understand, communicate, compute, and process drug-specific information in order to make informed medication and health decisions that ensure safe and effective medication use [5]. Evidence substantiated its positive correlation with medication adherence and blood pressure control in hypertensive populations [6–8]. In recent years, most studies on medication literacy employed a “variable-centered” approach, assuming sample homogeneity and focusing on group characteristics [9]. However, this approach overlooks individual differences and group heterogeneity, potentially masking distinct subpopulations with unique medication literacy patterns that require tailored interventions.

This study employs Bandura’s Social Cognitive Theory first articulated in 1986 [10], with particular emphasis on triadic reciprocal determinism and self-efficacy theory. Triadic reciprocal determinism conceptualizes environment, personal cognition, and behavior as interdependent components of a unified system. These elements interact dynamically, with behavior emerging as a product of the continuous interplay between cognitive processes and environmental contexts. Self-efficacy theory posits that individuals’ beliefs in their capabilities exert significant influence on behavioral outcomes. In the present study, medication literacy is operationalized as the behavioral component, social support as the environmental factor, and illness perception as the cognitive element.

Latent profile analysis (LPA), a “person-centered” method, identifies group heterogeneity by analyzing shared response patterns and classifying individuals into distinct subgroups [11,12]. Unlike traditional cluster analysis, LPA offers several advantages: it uses maximum likelihood estimation for greater statistical rigor, provides probabilistic class membership rather than deterministic classification, and offers comprehensive model fit indices to guide class enumeration. This approach improves classification accuracy and captures subgroup characteristics, which is useful for guiding individualized clinical practice and achieving improved intervention outcomes, particularly for vulnerable groups [13]. Unfortunately, the heterogeneity of medication literacy among hypertensive patients, especially among the young and middle-aged people has yet to be explored. Therefore, this study used the LPA to explore the latent profile characteristics of medication literacy, with a specific focus on association factors of each subgroup, to provide evidence for developing targeted intervention strategies.

## 2. Methods

### 2.1 Participants

This cross-sectional study enrolled 544 consecutively recruited hypertensive adults (aged 18–59 years) from two tertiary hospitals in a single-city of China between July 15 and November 15, 2024. The sample size was considered adequate based on Monte Carlo simulation studies for latent profile analysis [14]. The study protocol was approved by the Ethics Review Board of Huzhou University (No. 202240703, dated January 10, 2024). The inclusion criteria were as follows: (1) age 18–59 years, (2) confirmed hypertension diagnosis per 2024 Chinese Hypertension Management Guidelines [15], (3) current or recent (≤3 months) antihypertensive medication use, (4) adequate cognitive/communication capacity, and (5) written informed consent. Exclusion criteria included: (1) psychiatric comorbidities or severe hypertensive complications (e.g., encephalopathy, hypertensive crisis), (2) major systemic disorders (malignancy, recent cardiovascular/cerebrovascular events, end-stage renal disease), (3) secondary hypertension, and (4) participation in other hypertension trials within 30 days prior to the study.

### 2.2 Research tools and data collection

The study incorporated five validated instruments: (1) a Demographic and Disease-Related Characteristics Questionnaire, (2) the Revised Chinese Medication Literacy Scale for Hypertensive Patients (C-MLSHP-R), (3) the Perceived Social Support Scale (PSSS), (4) the General Self-Efficacy Scale (GSES), and (5) the Brief Illness Perception Questionnaire (BIPQ). The C-MLSHP-R (18 items) operationalizes medication literacy through four domains: medication knowledge, medication attitude, medication skills, and medication behaviors [16]. The medication knowledge dimension includes four multiple-choice items, with one point awarded for each correct answer. The medication attitude dimension consists of three items, and the medication behavior dimension comprises four items, both rated on a 5-point Likert scale (0–4 points). Items A1-A3 are reverse-scored. The medication skill dimension contains seven items, with one point assigned for a correct answer and zero points for an incorrect answer or a response of “do not know.” The total scale score ranges from 0 to 51 points, with higher scores indicating a higher level of medication literacy among patients with hypertension. The PSSS is a 12-item self-report inventory comprising three domains: family support, friend support, and other support [11]. All items were rated using a 7-point Likert scale. The total scale score ranges from 12 to 84, with higher scores indicating a higher level of social support among patients. The GSES questionnaire consists of 10 items measured on a 4-point Likert scale ranging from 1 (“completely disagree”) to 4 (“completely agree”) [17]. Scale scores range from 10 to 40. Higher scores reflect a more positive perception and evaluation of one’s own capabilities. The BIPQ includes three dimensions: disease cognition, emotion and disease understanding [18]. It consists of 9 items, including 3 reverse-scored items, 5 forward-scored items, and 1 open-ended question, rated on a 0–10 scale.The scale ranges from 0 to 80. Higher scores indicate more negative disease perceptions and emotions. The reliability of the scales was assessed using Cronbach’s α coefficient in this study. The Cronbach’s α was 0.903 for the C-MLSHP-R, 0.921 for the PSSS, 0.916 for the GSES, and 0.892 for the BIPQ, all above 0.8, indicating good to excellent internal consistency.

Participants were first informed about the confidentiality and content of the study. After providing written informed consent, participants voluntarily completed the questionnaires. Questionnaires were administered and returned on-site by five uniformly trained research assistants. For a small number of illiterate patients, a researcher read the content of the questionnaire aloud and completed it according to the patient’s wishes. The sample size for this study met the recommended threshold for cross-sectional analyses of this nature [14,19,20], which a minimum sample size of about 500 should lead to enough accuracy in identifying a correct number of latent profiles. Of the 573 questionnaires distributed, 29 were excluded as invalid, including 5 with missing data on core variables, 9 with obvious logical errors, and 15 with straight-lined responses (same answer for all items).

### 2.3 Data analysis

Data were analyzed using SPSS 26.0 and Mplus 8.3. The four domain scores of the Revised Chinese Medication Literacy Scale for Hypertensive Patients (C-MLSHP-R) were entered as indicators in latent profile analysis. Measurement data were described as means and standard deviations, while count data were described using frequencies and composition ratios. A two-step approach was employed for the regression analysis. First, bivariate analyses (Chi-square tests for categorical variables and ANOVA for continuous variables) were conducted to identify candidate variables associated with medication literacy profiles. Subsequently, variables demonstrating statistical significance (*P <* 0.05) in the bivariate analyses, along with theoretically relevant sociodemographic confounders, were simultaneously entered into a multivariate multinomial logistic regression model to identify independent association factors while controlling for potential confounding effects. These covariates included age, sex, place of residence, occupational/work-related status, blood pressure monitoring, education level, per capita monthly household income, PSSS, GSES, and BIPQ. All covariates were retained in the final model regardless of statistical significance. Before model fitting, multicollinearity among predictors was assessed. Because occupational status and type of work were structurally overlapping, they were handled as occupational/work-related status to avoid collinearity and unstable parameter estimates. The high medication literacy profile was used as the reference category. Prior to conducting the regression, multicollinearity among the predictor variables was assessed using the Variance Inflation Factor (VIF). All VIF values ranged from 1.029 to 3.056, falling well below the conventional threshold of 5, indicating that multicollinearity did not bias the parameter estimates. Latent profile modeling was conducted using the robust Maximum Likelihood Estimator (MLR) in Mplus 8.3 to account for potential non-normality in the continuous indicators. The models were specified such that variances were freely estimated across profiles, while covariances between indicators were constrained to zero within classes (local independence assumption). Modeling began with a baseline model (one class), gradually increasing the number of classes and evaluating the model fit indices for each. To avoid local maxima, 500 random starts with 100 final stage optimizations were employed, and the best log-likelihood value was verified to be successfully replicated across starts [19]. The fit indicator criteria included: (1) Akaike Information Criterion (AIC), Bayesian Information Criterion (BIC), and adjusted BIC (aBIC), where lower values indicate better fit; (2) entropy, where values closer to 1 indicate better classification accuracy; (3) Lo-Mendell-Rubin adjusted likelihood ratio test (LMR) and Bootstrap Likelihood Ratio Test (BLRT), with *P <* 0.05 indicating that a K-class model fits significantly better than a K-1 class model. All tests were performed using a two-sided with a significance level of 0.05.

## 3. Results

### 3.1 Demographic characteristics

Of the 573 questionnaires distributed, 544 valid questionnaires were included, with an effective response rate of 94.94%. No missing data were observed for all variables entered into latent profile analysis and multinomial logistic regression. Table 1 presented the general characteristics of the 544 young and middle-aged patients with hypertension.

**Table 1:** Demographic and disease characteristics of each latent profile (n = 544).

Variables	Overall (n = 544)	P1: low medication literacy	P2: moderate medication literacy	P3: high medication literacy	χ²/F	*P* value
		(n = 192)	(n = 273)	(n = 79)		
Gender						
Male	330 (60.7%)	115 (59.9%)	162 (59.3%)	53 (67.1%)	1.614	0.446
Female	214 (39.3%)	77 (40.1%)	111 (40.7%)	26 (32.9%)		
Age (year)						
18-40	140 (25.7%)	31 (16.1%)	84 (30.8%)	25 (31.6%)	28.011	<0.001
41-50	218 (40.1%)	73 (38.0%)	105 (38.5%)	40 (50.6%)		
51-59	186 (34.2%)	88 (45.8%)	84 (30.8%)	14 (17.7%)		
Education						
Junior middle school and below	324 (59.6%)	160 (83.3%)	149 (54.6%)	15 (19.0%)	103.077	<0.001
Senior school or technical secondary school	104 (19.1%)	19 (9.9%)	56 (20.5%)	29 (36.7%)		
Junior college/ College degree or above	116 (21.3%)	13 (6.8%)	68 (24.9%)	35 (44.3%)		
Residence						
Urban	378 (69.5%)	110 (57.3%)	203 (74.4%)	65 (82.3%)	22.620	<0.001
Rural areas	166 (30.5%)	82 (42.7%)	70 (25.6%)	14 (17.7%)		
Occupation status						
On-the-job	394 (72.4%)	116 (60.4%)	214 (78.4%)	64 (81.0%)	21.848	<0.001
Jobless	93 (17.1%)	46 (24.0%)	37 (13.6%)	10 (12.7%)		
Retired	57 (10.5%)	30 (15.6%)	22 (8.1%)	5 (6.3%)		
Work type						
Mainly manual labor	269 (49.4%)	109 (56.8%)	129 (47.3%)	31 (39.2%)	19.123	0.001
Mainly mental labor	236 (43.4%)	63 (32.8%)	127 (46.5%)	46 (58.2%)		
Neither	39 (7.2%)	20 (10.4%)	17 (6.2%)	2 (2.5%)		
Marital status						
Married	461 (84.7%)	168 (87.5%)	229 (83.9%)	64 (81.0%)	7.804	0.137^*^
Unmarried	62 (11.4%)	14 (7.3%)	37 (13.6%)	11 (13.9%)		
Divorced	18 (3.3%)	9 (4.7%)	6 (2.2%)	3 (3.8%)		
Widowed	3 (0.6%)	1 (0.5%)	1 (0.4%)	1 (1.3%)		
Medical insurance type						
Self-paying or other	51 (9.4%)	20 (10.4%)	27 (9.9%)	4 (5.1%)	7.927	0.244
New rural cooperative medical insurance	42 (7.7%)	21 (10.9%)	18 (6.6%)	3 (3.8%)		
Medical insurance for urban workers	402 (73.9%)	134 (69.8%)	205 (75.1%)	63 (79.7%)		
Medical insurance for urban residents	49 (9.0%)	17 (8.9%)	23 (8.4%)	9 (11.4%)		
The number of people who live together						
1 or below	130 (23.9%)	40 (20.8%)	73 (26.7%)	17 (21.5%)	2.450	0.294
2 or above	414 (76.1%)	152 (79.2%)	200 (73.3%)	62 (78.5%)		
Per capita monthly household income (¥)^**^						
<5,000	179 (32.9%)	114 (59.4%)	55 (20.1%)	10 (12.7%)	113.550	<0.001
5,000-10,000	230 (42.3%)	58 (30.2%)	142 (52.0%)	30 (38.0%)		
>10,000	135 (24.8%)	20 (10.4%)	76 (27.8%)	39 (49.4%)		
History of hypertension (year)						
<5	260 (47.8%)	82 (42.7%)	144 (52.8%)	34 (43.0%)	7.576	0.108
5-10	181 (33.3%)	67 (34.9%)	88 (32.2%)	26 (32.9%)		
>10	103 (18.9%)	43 (22.4%)	41 (15.0%)	19 (24.1%)		
History of antihypertensives (year)						
<5	303 (55.7%)	101 (52.6%)	163 (59.7%)	39 (49.4%)	5.780	0.216
5-10	149 (27.4%)	58 (30.2%)	70 (25.6%)	21 (26.6%)		
>10	92 (16.9%)	33 (17.2%)	40 (14.7%)	19 (24.1%)		
BP monitoring						
Yes	398 (73.2%)	100 (52.1%)	228 (83.5%)	70 (88.6%)	67.951	<0.001
No	146 (26.8%)	92 (47.9%)	45 (16.5%)	9 (11.4%)		
Types of medications						
1	362 (66.5%)	126 (65.6%)	181 (66.3%)	55 (69.6%)	2.901	0.574
2	147 (27.0%)	54 (28.1%)	71 (26.0%)	22 (27.8%)		
≥ 3	35 (6.4%)	12 (6.3%)	21 (7.7%)	2 (2.5%)		
Family history of hypertension						
Yes	332 (61.0%)	112 (58.3%)	173 (63.4%)	47 (59.5%)	1.294	0.524
No	212 (39.0%)	80 (41.7%)	100 (36.6%)	32 (40.5%)		
Medical background						
Yes	96 (17.6%)	28 (14.6%)	56 (20.5%)	12 (15.2%)	3.111	0.211
No	448 (82.4%)	164 (85.4%)	217 (79.5%)	67 (84.8%)		
Number of comorbidities						
0	328 (60.3%)	112 (58.3%)	164 (60.1%)	52 (65.8%)	2.664	0.850
1	165 (30.3%)	64 (33.3%)	81 (29.7%)	20 (25.3%)		
2	41 (7.5%)	13 (6.8%)	23 (8.4%)	5 (6.3%)		
≥3	10 (1.8%)	3 (1.6%)	5 (1.8%)	2 (2.5%)		
Side effect of medication						
Yes	203 (37.3%)	77 (40.1%)	94 (34.4%)	32 (40.5%)	1.952	0.377
No	341 (62.7%)	115 (59.9%)	179 (65.6%)	47 (59.5%)		
PSSS（mean±SD）	53.95 ± 10.73	46.03 ± 8.01	56.77 ± 9.39	63.51 ± 7.88	141.15	<0.001
GSES（mean±SD）	26.78 ± 5.60	22.84 ± 4.14	28.08 ± 5.13	31.85 ± 3.74	128.49	<0.001
BIPQ（mean±SD）	37.28 ± 12.53	47.54 ± 8.21	33.60 ± 10.78	25.06 ± 8.02	195.01	<0.001

* Fisher’s exact probability method.

** US$1 equals ¥7.1793, exchange rate on 1 February 2025.

PSSS, the Perceived Social Support Scale; GSES, the General Self-Efficacy Scale;

BIPQ, the Brief Illness Perception Questionnaire.

### 3.2 Latent profile analysis and characteristics of latent classes

**Fig 1:**
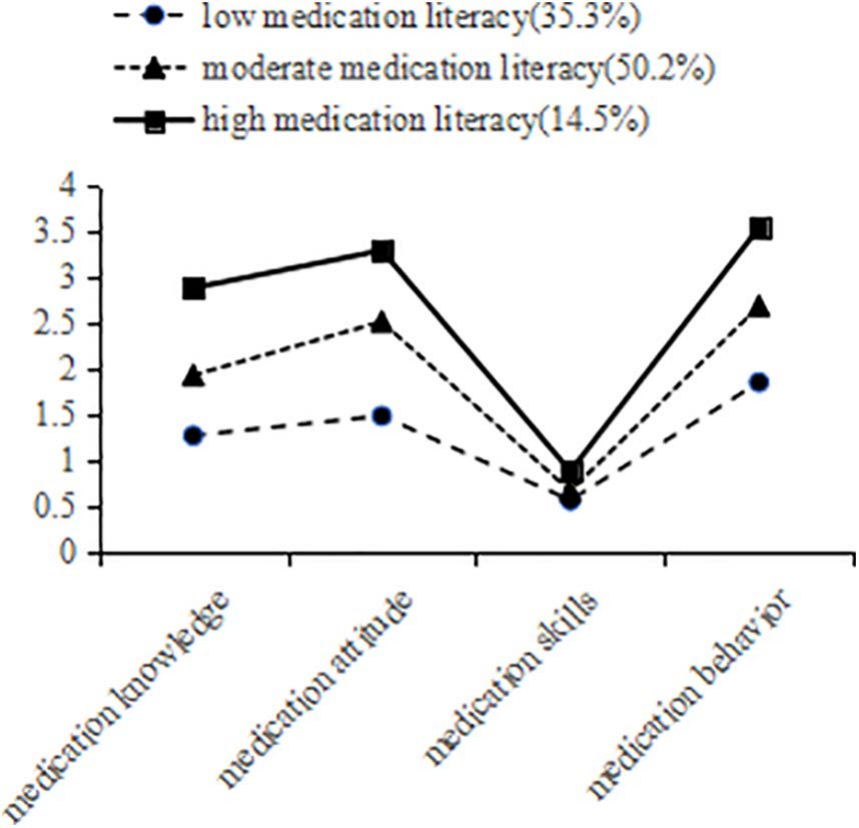
Mean scores for each dimension of the three-profile model of medication literacy (n = 544).

As shown in Table 2, while the AIC, BIC, and aBIC values continued to decrease with additional classes, model selection balanced statistical fit with parsimony, classification quality, and substantive interpretability [21]. The 4-profile model was not supported by the LMR test (*P* = 0.108), suggesting that the improvement in fit from three to four classes was not statistically significant. The 5-profile model yielded a class containing only 0.9% of the sample, below the recommended 5% threshold raising concerns about overfitting [21,22]. In contrast, the 3-profile model demonstrated excellent classification accuracy (entropy = 0.927; average posterior probabilities of 97.2%, 96.7%, and 97.9%, all exceeding 90%) and yielded theoretically coherent and clinically interpretable profiles. Therefore, the 3-profile solution was selected as the optimal model. The characteristics of the latent classes were detailed in Fig 1 and Table 3. Profile 1 (n = 192, 35.3%), characterized by consistently low scores across all items, was designated as “low medication literacy (Low C-MLSHP-R)”. Profile 2 (n = 273, 50.2%) exhibited moderate performance across domains, classified as “moderate medication literacy (moderate C-MLSHP-R)”. Profile 3 (n = 79, 14.5%) demonstrated uniformly high item scores, identified as “high medication literacy (High C-MLSHP-R)”.

**Table 2:** Fit statistics for profile structure (n = 544).

No. of profiles	AIC	BIC	aBIC	Entropy	LMR	BLRT	Latent profile proportion (%)
1	2915.486	2949.878	2924.483	^__^	^__^	^__^	^__^
2	2167.036	2222.922	2181.655	0.789	0.008	<0.001	0.546/0.454
3	1585.879	1663.260	1606.121	0.927	<0.001	<0.001	0.353/0.502/0.145
4	1493.893	1592.769	1519.758	0.936	0.108	<0.001	0.357/0.143/0.053/0.447
5	1437.835	1558.206	1469.323	0.945	<0.001	<0.001	0.449/0.355/0.009/0.053/0.134

aBIC, adjusted BIC; AIC, Akaike information criterion; BIC, Bayesian information criterion; BLRT, bootstrap likelihood ratio test; LMR, Lo-Mendell-Rubin likelihood ratio test.

**Table 3:** Mean scores and standard deviation for each dimension of the three-profile model of medication literacy (n = 544).

	Profile 1	Profile 2	Profile 3
	Low medication literacy (n = 192)	Moderate medication literacy (n = 273)	High medication literacy (n = 79)
	M (SD)	M (SD)	M (SD)
Medication knowledge	1.26 ± 0.04	1.93 ± 0.02	2.88 ± 0.03
Medication attitude	1.47 ± 0.04	2.52 ± 0.03	3.30 ± 0.09
Medication skills	0.56 ± 0.01	0.66 ± 0.01	0.88 ± 0.01
Medication behavior	1.85 ± 0.02	2.68 ± 0.02	3.55 ± 0.02

M = Mean; SD = Standard deviation.

### 3.3 Association factors of medication literacy

Group comparisons identified differences in age, educational level, residence, occupational status, work type, per capita monthly household income, blood pressure monitoring, as well as PSSS, GSES, and BIPQ scores (e.g., Table 1). The latent profile membership (low, moderate, or high medication literacy) was used as the dependent variable. Demographic information, disease-related characteristics, variables that were statistically significant in bivariate analyses, and PSSS, GSES, and BIPQ scores were entered into a multinomial logistic regression model. The coding of the independent variables was detailed in Table 4. After adjustment for age, sex, place of residence, occupational/work-related status, blood pressure monitoring, education level, per capita monthly household income, PSSS, GSES, and BIPQ, several factors remained associated with medication literacy profiles (e.g., Table 5). Compared with the high medication literacy profile, patients with lower educational attainment, lower income, lower perceived social support, lower self-efficacy, and higher illness perception scores were more likely to be classified into the low medication literacy profile. The fully adjusted model also allowed assessment of non-significant covariates, which are now reported in Table 5. Compared to patients with a college degree or above (reference group), those with junior high school education or below had 6.55 times higher odds of belonging to the low medication literacy profile (OR = 6.553, 95% CI: 1.916–22.408) and 2.30 times higher odds of belonging to the moderate medication literacy profile (OR = 2.303, 95% CI: 1.042–5.093). Similarly, patients with a per capita monthly household income below 5,000 RMB had 3.84 times higher odds of low medication literacy compared to those earning above 10,000 RMB (OR = 3.842, 95% CI: 1.122–13.147). Each one-unit increase in social support (PSSS) was associated with a 12.3% reduction in the odds of low medication literacy (OR = 0.877, 95% CI: 0.835–0.921), and each one-unit increase in self-efficacy (GSES) was associated with an 18.8% reduction (OR = 0.812, 95% CI: 0.734–0.899). Conversely, each one-unit increase in negative illness perception (BIPQ) was associated with a 13.5% increase in the odds of low medication literacy (OR = 1.135, 95% CI: 1.081–1.192). It should be noted that some OR estimates (e.g., education: junior middle school and below) exhibited wide confidence intervals due to sparse cell counts in the high medication literacy profile (n = 79). These estimates should be interpreted with caution, and the direction of association rather than the precise magnitude is more informative in these cases.

**Table 4:** Assignment of independent variables in multiple logistic regression.

Variables	Assignment of variables
Latent categories	Low medication literacy = 1, moderate medication literacy = 2, high medication literacy = 3
Age	18-40 years = 1, 41–50 years = 2, 51–59 years = 3
Degree of education	Junior high school and below=1, technical secondary school/senior high school = 2, junior college/college degree and above = 3
Residence	Urban = 1, rural = 2
Occupational status	Employed = 1, unemployed or jobless = 2, retired = 3
Type of work	Mainly manual labor = 1, mainly mental labor = 2, neither = 3
Average monthly household income	<5,000 RMB = 1, 5,000-10,000 RMB = 2,>10,000 RMB = 3
Monitored blood pressure	Monitored = 1, not monitored = 2

**Table 5:** Multinomial logistic regression analysis of association factors (n = 544).

Variable	High medication literacy (Ref)					
	Low medication literacy			Moderate medication literacy		
	OR	95%CI	*P* Value	OR	95%CI	*P* Value
Age 41–50 years (Ref: 18–40 years)	0.239	0.072-0.797	0.020	0.153	0.063-0.375	<0.001
Age 51–59 years (Ref: 18–40 years)	0.361	0.079-1.659	0.191	0.146	0.040-0.531	0.003
Education（Ref: Junior college/ College degree or above）						
Junior middle school and below	6.553	1.916-22.408	0.003	2.303	1.042-5.093	0.039
Senior school or technical secondary school	3.316	0.848-12.967	0.085	0.951	0.473-1.909	0.887
Residence: rural (Ref: urban)	0.382	0.104-1.406	0.148	0.180	0.056-0.581	0.004
Occupational status: jobless (Ref: on-the-job)	36618831.4	0.000-Inf	0.995	6479488.7	0.000-Inf	0.995
Occupational status: retired (Ref: on-the-job)	1.888	0.139-25.625	0.633	1.251	0.107-14.635	0.858
Per capita monthly household income（Ref: > 10,000 RMB）						
5,000 RMB	3.842	1.122-13.147	0.032	0.919	0.352-2.399	0.862
5,000–10,000 RMB	2.307	0.818-6.509	0.114	1.536	0.799-2.950	0.198
BP monitoring: No (Ref: Yes)	9.843	1.322-73.272	0.026	2.952	0.422-20.658	0.275
PSSS	0.877	0.835-0.921	<0.001	0.963	0.927-1.000	0.049
GSES	0.812	0.734-0.899	<0.001	0.887	0.818-0.962	0.004
BIPQ	1.135	1.081-1.192	<0.001	1.047	1.008-1.087	0.017

Note: High medication literacy was used as the reference group. Model adjusted for age, sex, residence, occupational status, blood pressure monitoring, education, income, PSSS, GSES, and BIPQ. Type of work was not simultaneously included because it was collinear with occupational status in the SAV. ORs for jobless and income <5,000 are unstable because the high-literacy group had zero cases in those categories. Consider category merging or penalized multinomial logistic regression.

PSSS, the Perceived Social Support Scale; GSES, the General Self-Efficacy Scale.

BIPQ, the Brief Illness Perception Questionnaire.

### 3.4 Sensitivity analysis

Split-half cross-validation confirmed the stability of the 3-profile solution. In both random subsamples (n₁ = 272, n₂ = 272), the 3-profile model was identified as optimal based on the same criteria applied to the full sample. The models exhibited excellent classification accuracy in both subsamples (entropy = 0.904 and 0.920, respectively). More importantly, the tripartite structure (low, moderate, and high medication literacy) was successfully replicated, with profile proportions and domain-specific mean score patterns closely mirroring those of the full-sample analysis. These findings indicate that the 3-profile solution is robust and not an artifact of sample-specific characteristics.

## 4. Discussion

Medication literacy is a key factor in determining the use of health-related information, promoting health, and improving the quality of life. However, in China, young and middle-aged patients with hypertension often exhibit suboptimal medication literacy and adherence [23,24]. Unlike previous investigations [25,26], this study is among the first to apply a person-centered approach and empirically focused on the characteristics of medication literacy among young and middle-aged Chinese hypertensive patients, thereby complementing the limitations of previous studies and offering a novel perspective for future research on medication literacy.

This study revealed that the medication literacy score for young and middle-aged hypertensive patients was 28.78 ± 7.58, suggesting an intermediate level with significant potential for improvement. While scores marginally exceeded those reported in prior research [27], the discrepancy likely reflects differences in sample characteristics across studies (e.g., age range, regional healthcare access). Meanwhile, latent profile analysis identified three distinct medication literacy profiles: low (35.3%), moderate (50.2%), and high (14.5%) groups, establishing a tripartite classification of patient capabilities. Also, significant intergroup variations emerged across educational level, per capita monthly family income, PSSS, GSES, and BIPQ scores (Tables 1–3, Fig 1), confirming population heterogeneity. These findings deepen the understanding of medication literacy as a multifaceted construct and provide an empirical basis for tailoring interventions to specific patient subgroups. Consistent with Social Cognitive Theory, the identified medication literacy profiles suggest that medication literacy extends beyond knowledge acquisition. It reflects a dynamic interplay among cognitive factors (self-efficacy, illness perception), environmental influences (social support), and behavioral determinants. These profile-specific characteristics suggest tailored intervention strategies: low-literacy patients may benefit from foundational knowledge and skills training, moderate-literacy patients require reinforcement of positive attitudes and behaviors, and high-literacy patients could serve as peer educators or mentors. This theoretically grounded stratification moves beyond descriptive classification by highlighting specific pathways that could inform future intervention design. However, these recommendations are preliminary and hypothesis-generating, rather than clinical guidelines.

In this study, patients with junior high school or below were significantly more likely to be categorized into low medication literacy groups (OR = 6.553) compared to their counterparts with higher educational attainment (e.g., Table 5). Consistent with research in other populations [28], lower education levels were associated with greater delays in seeking care and reduced participation in cardiovascular health programs. These findings align with previous studies showing that higher educational attainment correlates with better medication knowledge, more positive attitudes, and improved medication practices [29]. To address these educational disparities, tailored approaches such as patient-centered visual tools (e.g., graphical medication schedules, 3D animated vascular models) and short video-based instruction may help optimize knowledge retention in this population [30,31].

The study also found that individuals with monthly household incomes below 5,000 RMB demonstrated a significantly higher propensity for classification into the low medication literacy (OR = 3.842), highlighting their close association with medication literacy in these hypertensive patients. One possible explanation is that people with high incomes are willing to spend a lot of money on health care and health promotion and generally have a high educational level. This finding is consistent with other studies conducted in China which found that socioeconomically advantaged patients exhibited reduced psychosocial stressors but enhanced health maintenance behaviors (such as proactive engagement in preventive healthcare measures, increased frequency of clinical consultations and utilization of premium healthcare services) [32]. These findings underscored the imperative for nursing professionals to integrate socioeconomic status evaluations into comprehensive care frameworks.

Aligned with a longitudinal analysis [33], this study also substantiated the social support systems was an association factor with medication literacy in hypertensive patients. In China, due to the intense pressure of work among young and middle-aged populations, which often brings challenges such as reduced social opportunities, loss of social roles. Therefore, these people also lack time and energy to care for them, their social support level will be further reduced. However, optimal social support provision facilitates both emotional comfort and health information accessibility, thereby fostering the development of medication literacy through dual channels. In addition, strengthening the support networks within families, such as enhancing emotional care and providing practical assistance from children and relatives, may provide significant protection against low medication literacy. Thus, nursing staff should implement routine social support assessments and develop family inclusive care protocols to optimize therapeutic alliances and medication literacy outcomes.

Consistent with prior studies [17,18,34], this study found a significant positive correlation between patients’ self-efficacy scores and their medication literacy levels. Higher self-efficacy may foster a more resolute problem-solving attitude and better resistance to the negative impacts of health-related challenges. This also suggests that higher self-efficacy means patients gain a clearer understanding of their condition, feel a stronger sense of control, and find greater meaning in life, thereby promoting an improvement in medication literacy. Based on these findings, clinical nurses could consider strategically integrate self-efficacy assessments into routine health education protocols in these hypertensive patients. Encouraging patients with higher self-efficacy to serve as role models for those with lower self-efficacy may be beneficial. Additionally, helping patients establish milestones and regularly reflect on their disease experiences could further enhance self-efficacy and medication literacy.

The findings of this study are consistent with those of previous studies that showed negative correlation between disease perception scores and medication literacy levels [35,36]. Higher levels of medication literacy facilitate enhanced understanding of disease potential risks and consequences, the knowledge and capabilities to take appropriate actions, thereby fostering a more objective and rational illness perception. At the same time, disease self-perception can provide information to formulators of public health policies so that they can take measures aimed at encouraging the practice, especially with regard to the low medication literacy of young and middle-aged hypertensive populations. The observed associations among social support, self-efficacy, illness perception, and medication literacy profiles are broadly consistent with Social Cognitive Theory. However, these cross-sectional associations do not establish causal directionality. This design precludes determination of whether these factors precede and influence medication literacy, whether medication literacy shapes these psychosocial attributes, or whether unmeasured confounders account for these relationships. For instance, low medication literacy may lead to suboptimal disease control, subsequently diminishing self-efficacy and amplifying negative illness perceptions. Longitudinal studies are warranted to elucidate these temporal relationships and empirically test the proposed theoretical model.

These findings highlight the potential value of structured psychological evaluation protocols for routine nursing assessments. Specifically, attention could be given to patients with high illness perception scores, with the goal of alleviating negative emotions and fostering a more accurate understanding of their condition—potentially through cognitive behavioral therapy or structured educational interventions. Nurses may also encourage patients to take a more active role in learning about their medications as part of routine care.

**Limitations:** This study provided valuable insights, but several limitations should be acknowledged. First, a small sample size and the exclusive recruitment of participants from a single city in China created geographical homogeneity and limited the generalizability, as regional variations in socioeconomic status, cultural practices, and healthcare infrastructure may influence medication literacy patterns. To enhance the generalizability and applicability of the findings, future studies should expand the participant pool to include hypertensive patients from different provinces and diverse cultural contexts in China. Second, the cross-sectional study design inherently precludes the establishment of causal relationships between variables and only verifies their associative relationships. Additionally, while the 3-profile model was identified as optimal, and the high entropy and posterior probabilities of our model mitigate classification error, the classify-then-analyze approach used in our regression models may still introduce some degree of bias in parameter estimates. Third, a small number of illiterate patients had their questionnaires completed by researchers following verbal administration, which may have introduced measurement bias or social desirability bias. Prospective longitudinal studies are warranted in the future to clarify the temporal relationships between variables and adopt more objective evaluation indicators to reduce the impact of bias on study results. Fourth, the relatively small sample size of the high medication literacy profile (n = 79) limited the number of covariates that could be reliably included in the multinomial logistic regression model. The sparse data in this subgroup led to wide confidence intervals for some OR estimates, particularly for education and income categories. Future studies with larger sample sizes are needed to provide more precise estimates and to allow for comprehensive covariate adjustment including demographic confounders such as age, sex, and place of residence. Finally, the focus on young and middle-aged hypertension patients limited the understanding of developmental trajectories in medication management competencies. More diverse samples are necessary for broader applicability of the results.

## 5. Conclusion

Overall, this study identify distinct subgroup characteristics of medication literacy among young and middle-aged Chinese patients with hypertension. This classification also provide evidence of the heterogeneity of medication literacy within this population. Associated factors include education level, per capita monthly household income, social support, self-efficacy, and illness perception. These findings extend Social Cognitive Theory to medication literacy research by illustrating how personal, environmental, and behavioral factors differentiate patient subgroups, thereby contributing to the theoretical understanding of medication literacy development in the context of chronic disease. These findings may inform the development of tailored intervention programs aimed at enhancing medication literacy among young and middle-aged patients with hypertension. However, given the observational design and the methodological constraints discussed above, these recommendations should be regarded as preliminary and hypothesis-generating rather than evidence-based clinical guidelines. In practice, individualized interventions may be most beneficial when tailored to patients’ medication literacy profiles. Particular attention may be given to patients with low medication literacy, with a focus on associated factors such as education level, household income, social support, self-efficacy, and illness perception.

## References

[1] NCD Risk Factor Collaboration (NCD-RisC). Worldwide trends in hypertension prevalence and progress in treatment and control from 1990 to 2019: a pooled analysis of 1201 population-representative studies with 104 million participants. Lancet. 2021;398(10304):957–80. doi: 10.1016/S0140-6736(21)01330-1 34450083 PMC8446938

[2] Liu Y, Jiang F, Zhang M, Niu H, Cao J, Du S, et al. Health literacy and self-management among middle-aged and young hypertensive patients: a parallel mediation effect of illness perception and self-efficacy. Front Psychol. 2024;15:1349451. doi: 10.3389/fpsyg.2024.1349451 38765827 PMC11099212

[3] Luo D, Cheng Y, Zhang H, Ba M, Chen P, Li H, et al. Association between high blood pressure and long term cardiovascular events in young adults: systematic review and meta-analysis. BMJ. 2020;370:m3222. doi: 10.1136/bmj.m3222 32907799 PMC7478061

[4] Li S, Xu T, Wen H, Guo Y. Prevalence, numbers and mortality risk of hypertensive patients with depressive symptom in China. BMC Cardiovasc Disord. 2025;25(1):92. doi: 10.1186/s12872-025-04536-8 39934667 PMC11816983

[5] Pouliot A, Vaillancourt R, Stacey D, Suter P. Defining and identifying concepts of medication literacy: an international perspective. Res Social Adm Pharm. 2018;14(9):797–804. doi: 10.1016/j.sapharm.2017.11.005 29191647

[6] Al-Noumani H, Wu J-R, Barksdale D, Knafl G, AlKhasawneh E, Sherwood G. Health beliefs and medication adherence in Omanis with hypertension. J Cardiovasc Nurs. 2018;33(6):518–26. doi: 10.1097/JCN.0000000000000511 30130358 PMC6179908

[7] Lee S, Jeong K-H, Lee S, Park H. A study on types of medication adherence in hypertension among older patients and influencing factors. Healthcare (Basel). 2022;10(11):2322. doi: 10.3390/healthcare10112322 36421646 PMC9690494

[8] Ruksakulpiwat S, Schiltz NK, Irani E, Josephson RA, Adams J, Still CH. Medication adherence of older adults with hypertension: a systematic review. Patient Prefer Adherence. 2024;18:957–75. doi: 10.2147/PPA.S459678 38737487 PMC11088410

[9] Gentizon J, Hirt J, Jaques C, Lang P-O, Mabire C. Instruments assessing medication literacy in adult recipients of care: a systematic review of measurement properties. Int J Nurs Stud. 2021;113:103785. doi: 10.1016/j.ijnurstu.2020.103785 33080478

[10] Bandura A. Human agency in social cognitive theory. Am Psychol. 1989;44(9):1175–84. doi: 10.1037/0003-066x.44.9.1175 2782727

[11] Ding Y, Zhang H, Hu Z, Sun Y, Wang Y, Ding B, et al. Perceived social support and health-related quality of life among hypertensive patients: a latent profile analysis and the role of delay discounting and living alone. Risk Manag Healthc Policy. 2024;17:2125–39. doi: 10.2147/RMHP.S476633 39246592 PMC11380862

[12] Shao YJ, Duan XC, Xu XJ, Guo HY, Zhang ZY, Zhao S, et al. Latent profile and determinants of self-management behaviors among older adult patients with chronic diseases: a cross-sectional study. Front Public Health. 2025;13:1506545. doi: 10.3389/fpubh.2025.1506545 39975786 PMC11835868

[13] Băjenaru L, Balog A, Dobre C, Drăghici R, Prada G-I. Latent profile analysis for quality of life in older patients. BMC Geriatr. 2022;22(1):848. doi: 10.1186/s12877-022-03518-1 36368920 PMC9652949

[14] Tein J-Y, Coxe S, Cham H. Statistical power to detect the correct number of classes in latent profile analysis. Struct Equ Modeling. 2013;20(4):640–57. doi: 10.1080/10705511.2013.824781 24489457 PMC3904803

[15] Liu J. Highlights of the 2024 Chinese hypertension guidelines. Hypertens Res. 2025;48(3):1048–53. doi: 10.1038/s41440-024-02070-2 39762483

[16] Qin N, Duan Y, Yao Z, Shi S, Liu H, Li X, et al. Psychometric properties and validation of the revised Chinese Medication Literacy Scale for Hypertensive Patients (C-MLSHP-R). Front Cardiovasc Med. 2022;9:976691. doi: 10.3389/fcvm.2022.976691 36148050 PMC9486212

[17] Ma G, Zhou C, Han Z, Mu T, Ma X. Social support and physical literacy in young and middle-aged patients with hypertension: the mediating effects of sense of coherence and self-efficacy. BMC Psychiatry. 2024;24(1):494. doi: 10.1186/s12888-024-05935-5 38978037 PMC11232136

[18] Lu T, Yang Z, Chen P, Li J, Zheng C, Kong L, et al. Influencing factors of medication literacy among community-dwelling older adult patients with hypertension: a study based on social learning theory. Front Pharmacol. 2023;14:1184701. doi: 10.3389/fphar.2023.1184701 37332350 PMC10272614

[19] Spurk D, Hirschi A, Wang M, Valero D, Kuroun S. Latent profile analysis: a review and “how to” guide of its application within vocational behavior research. J Vocat Behav. 2020;120:103445. doi: 10.1016/j.jvb.2020.103445

[20] Finch WH, Bronk KC. Conducting confirmatory latent class analysis using Mplus. Struct Equ Modeling. 2011;18(1):132–51. doi: 10.1080/10705511.2011.532732

[21] Sinha P, Calfee CS, Delucchi KL. Practitioner’s guide to latent class analysis: methodological considerations and common pitfalls. Crit Care Med. 2021;49(1):e63–79. doi: 10.1097/CCM.0000000000004710 33165028 PMC7746621

[22] Nylund KL, Asparouhov T, Muthén BO. Deciding on the Number of classes in latent class analysis and growth mixture modeling: a monte carlo simulation study. Struct Equ Modeling. 2007;14(4):535–69. doi: 10.1080/10705510701575396

[23] Pan Y, Li Y, Li Z, Zhou H, Zhou H, Wei Z, et al. A nomogram-based analysis on medication adherence of hypertensive patients in China. Asian Nurs Res (Korean Soc Nurs Sci). 2025;19(2):170–7. doi: 10.1016/j.anr.2025.01.008 40015669

[24] Ming X, Lu A-P, Liu Y-Y, Ju Y, Tian Q-Q, Tan X-H, et al. The status of medication literacy in young patients with hypertension and its relationship with medication adherence. J Cardiovasc Nurs. 2026;41(1):E18–24. doi: 10.1097/JCN.0000000000001214 40233009

[25] Ferreira PD, Simoes JA, Velho DC. Adherence to antihypertensive therapy and its determinants: a systematic review. Cureus. 2024;16(5):e59532. doi: 10.7759/cureus.59532 PMC11144025 38826951

[26] Loreto L, Linares-Jimenez FG, de Zeeuw J, de Winter AF. Health literacy and hypertension-related multimorbidity: unravelling the mediating role of self-management-insights from the lifelines cohort study. BMC Public Health. 2025;25(1):1530. doi: 10.1186/s12889-025-22798-x 40275236 PMC12020009

[27] Qin N, Yao Z, Shi S, Duan Y, Li X, Liu H, et al. Association between medication literacy and blood pressure control among hypertensive patients. Int J Nurs Pract. 2024;30(2):e13153. doi: 10.1111/ijn.13153 37062986

[28] Zhong Z, Zheng F, Guo Y, Luo A. Medication literacy in a cohort of Chinese patients discharged with acute coronary syndrome. Int J Environ Res Public Health. 2016;13(7):720. doi: 10.3390/ijerph13070720 27428990 PMC4962261

[29] Maluwa C, Kapira S, Chuljerm H, Parklak W, Kulprachakarn K. Impact of health education on knowledge retention among caregivers of hypertensive patients: a prospective cross-sectional study in rural Malawi. PLoS One. 2025;20(2):e0317684. doi: 10.1371/journal.pone.0317684 39899486 PMC11790085

[30] Ma G, Luo A, Shen Z, Duan Y, Shi S, Zhong Z. The status of medication literacy and associated factors of hypertensive patients in China: a cross-sectional study. Intern Emerg Med. 2020;15(3):409–19. doi: 10.1007/s11739-019-02187-0 31650433 PMC7165129

[31] Krychtiuk KA, Lopes RD, Cargill VA, Chen R, Cowie MR, Cushman WC, et al. Overcoming barriers to developing and implementing novel therapies for hypertension. Hypertension. 2025;82(10):1599–611. doi: 10.1161/HYPERTENSIONAHA.125.24992 40874957

[32] Li C, He D, Liu Y, Yang C, Zhang L. Anti-hypertensive medication adherence, socioeconomic status, and cognitive aging in the Chinese community-dwelling middle-aged and older adults ≥ 45 years: a population-based longitudinal study. BMC Med. 2025;23(1):121. doi: 10.1186/s12916-025-03949-8 40001139 PMC11863513

[33] Shen Z, Ding S, Shi S, Zhong Z. Association between social support and medication literacy in older adults with hypertension. Front Public Health. 2022;10:987526. doi: 10.3389/fpubh.2022.987526 36419989 PMC9677095

[34] Chan SW-C. Chronic disease management, self-efficacy and quality of life. J Nurs Res. 2021;29(1):e129. doi: 10.1097/JNR.0000000000000422 33427791 PMC7808345

[35] Alcântara L, Figueiredo T, Costa E. Exploring the perceptions and self-perceptions of therapeutic adherence in older adults with chronic conditions: a scoping review. Patient Prefer Adherence. 2025;19:503–26. doi: 10.2147/PPA.S496707 40046563 PMC11881769

[36] Gutierrez MM, Sakulbumrungsil R. Factors associated with medication adherence of hypertensive patients in the Philippines: a systematic review. Clin Hypertens. 2021;27(1):19. doi: 10.1186/s40885-021-00176-0 34593047 PMC8485436

