# Supplementary material for: Profiles of medication literacy and associated factors among young and middle-aged hypertensive patients in China: A latent profile analysis
Source: PLoS One. 2026 Jul 22;21(7):e0352857. doi: 10.1371/journal.pone.0352857 (PMC13390851; doi:10.1371/journal.pone.0352857)
Supplement: Attachment — Submitted filename: Response to reviewers.doc [file pone.0352857.s002.doc]

Dear the academic editor and Reviewers:

Thank you for your letter and for the reviewers’ comments concerning our manuscript entitled “Profiles of medication literacy and its predictors among young and middle-aged hypertensive patients in China: a latent profile analysis (PONE-D-25-50007)”. Those comments are all valuable and very helpful for revising and improving our paper, as well as the important guiding significance to our researches. We have considered all issues mentioned in the reviewers' comments carefully and have made correction which we hope meet with approval. Revised portion are marked in blue in the revised manuscript with track changes paper. The main corrections in the paper and the responses to the reviewer’s comments are as following:

**Responses to the academic editor’s comments:**

1. The manuscript presents a timely and methodologically sound application of latent profile analysis to explore heterogeneity in medication literacy among young and middle-aged patients with hypertension. The person-centred analytical approach is a clear strength and offers added value beyond traditional variable-centred methods. However, in its current form, the manuscript requires substantial refinement to ensure interpretive caution, conceptual clarity, and stronger translational relevance.

**Response:** Special thanks to you for your good comments. We have checked our manuscript carefully again, re-edited the title, corrected some vaguely expressed sentences in the manuscript to ensure interpretive caution, conceptual clarity, and stronger translational relevance. We think these changes will not influence the content and framework of the paper. Revised portion are marked in blue in the revised manuscript with track changes paper.

2. The primary areas requiring revision relate to how the study scope and findings are framed and interpreted. The manuscript would benefit from clearer definition and justification of the target population early in the Introduction, along with a more explicitly articulated research focus that guides the reader through the study rationale.

**Response:** Special thanks to you for your good comments. In the Introduction, we have supplemented the reasons for selecting the target population, along with a more explicitly articulated research focus. Revised portions are all marked in blue.

3. While the analytical methods are appropriate, several sections of the Results and Discussion employ language that may be interpreted as implying causality, despite the cross-sectional design. A more cautious interpretive stance is needed, particularly when describing associations between medication literacy profiles and sociodemographic or psychosocial variables.

**Response:** Special thanks to you for your good comments. We have checked our manuscript carefully again, corrected some vaguely expressed sentences in the manuscript to ensure interpretive caution, conceptual clarity. Revised portion are marked in blue in the revised manuscript with track changes paper.
4. Greater transparency is also required regarding the generalisability of the findings. The exclusive recruitment from tertiary hospitals in an economically developed region, combined with the focus on a single age group and reliance on self-reported measures, constrains the extent to which the results can be extrapolated to broader populations. These issues should be addressed more explicitly in the Limitations section and reframed as opportunities for future research, including longitudinal designs and recruitment across diverse geographic and healthcare contexts.

**Response:** Special thanks to you for your good comments. In this manuscript, we have supplemented the limitations, especially the homogeneous sample and reliance on self-reported data affect interpretation and generalizability. At the same time, we have pointed out a clear direction for future research. Revised portion are marked in blue in the revised manuscript with track changes paper.

**Response to Journal requirements:**

1. Please ensure that your manuscript meets PLOS ONE's style requirements, including those for file naming.

**Response:** We have checked our manuscript carefully again to ensure that our manuscript meets PLOS ONE's style requirements. Revised portion are marked in blue in the revised manuscript with track changes paper.

2. We noticed you have some minor occurrence of overlapping text with the following previous publication(s), which needs to be addressed.

**Response:** We have checked our manuscript carefully again to rephrase any duplicated text outside the methods section.

3. We note that the grant information you provided in the Funding Information and Financial Disclosure sections do not match.

**Response:** We have corrected the Financial Disclosure sections.

4. Please state what role the funders took in the study.

**Response:** We have declared the role of Funder statement. The funder role is writing the original draft and investigation.

5. We note that you have provided funding information that is currently declared in your Funding Statement. However, funding information should not appear in the Acknowledgments section or other areas of your manuscript.

**Response:** We have removed any funding-related text from the manuscript. Currently, Our Funding Statement reads as follows: “This study was funded by Natural Science Foundation of Huzhou city, Zhejiang province, China (2022YZ44).”

6. Your ethics statement should only appear in the Methods section of your manuscript. If your ethics statement is written in any section besides the Methods, please delete it from any other section.

**Response:** The ethics statement is written in the Methods and deleted from any other section.

**Responses to the reviewer’s comments:**

**Reviewer #1:**

Much appreciated for giving me the opportunity for this review, this study innovatively uses latent class analysis to explore group heterogeneity in drug literacy, going beyond the limitations of traditional variable-centric analysis. This “Human-centered” approach can more truly reflect the diversity and complexity of the patient population, and has important methodological value for developing precise and personalized clinical intervention strategies. But there are some areas for improvement, which should be more fully addressed in the “Limitations” of the discussion section, it is pointed out that the conclusions of this study are mainly applicable to patients in tertiary hospitals in economically developed areas similar to Zhejiang province in China. In conclusion or future research directions, it is suggested that follow-up studies should recruit samples from more diverse areas (such as Midwestern Sectional Figure Skating Championships, urban and rural communities) to verify the generalizability of the findings of this study. More cautious language should be used when discussing partial interpretation of the results, describing “Influencing factors” or “Predictors” as “Relevant factors” or “Association factors”, avoid words that imply causality. The overall language of the manuscript is fluent, but there are minor grammatical problems or repetitions in individual sentences. It is recommended that the final version be thoroughly polished in English. For example, the abstract and the introduction are somewhat repetitive and could be further refined.

**Response:** Special thanks to you for your good comments. We have checked our manuscript carefully again, corrected some vaguely expressed sentences in the manuscript to ensure interpretive caution, conceptual clarity. We also fully addressed the deficiencies of this study in the “Limitations” of the discussion section and revised minor grammatical problems. Revised portion are marked in blue in the revised manuscript with track changes paper.

**Reviewer #2:**
1. Introduction Review: The introduction is clear; however, defining young and middle-aged patients at the outset would help readers. In addition, briefly clarifying the research gap and stating a clear research question would make the study’s focus easier to follow.

**Response:** Special thanks to you for your good comments. In the introduction, we have checked our manuscript carefully again, more clearly indicated the research population, research purpose, and novelty to attract readers. Revised portion are marked in blue in the revised manuscript with track changes paper.
2. Methodology Review: The study is valuable and provides useful findings; however, a few points could be clarified to improve transparency. As the study uses a cross-sectional design, the results reflect associations only and do not allow for causal interpretations. Moreover, reliance on self-reported questionnaires means that some responses may not fully reflect patients’ actual experiences or behaviors. Focusing on one age group and a single geographic region may also limit the generalizability of the findings. Clearly acknowledging these points in the limitations section would strengthen the manuscript.

**Response:** Special thanks to you for your good comments. As the reviewer mentioned, the results reflect associations only, some responses may not fully reflect patients’ actual experiences or behaviors. In this manuscript, some imprecise expressions have been revised, and clearly acknowledging these points in the limitations section. Revised portion are marked in blue in the revised manuscript with track changes paper.
3. Results Review: The study is well conducted; however, the choice of the number of profiles involves some degree of judgment. Therefore, the findings should be interpreted with caution, particularly given the cross-sectional design and limited sample. Acknowledging the potential impact of self-reported data on result accuracy would further strengthen the results section.

**Response:** Special thanks to you for your good comments. In this study, the sample size was in accordance with the recommended sample size for potential cross-sectional analysis, specifically n>500 [References17]. And 573 questionnaires distributed, 29 were excluded as invalid. We also presented the findings with caution. However, this application requires further investigation. Future research should prioritize longitudinal studies to establish temporal relationships and causality. And we also clearly acknowledging these insufficient points in the limitations section. Revised portion are marked in blue in the revised manuscript with track changes paper.
4. Discussion Review: The discussion is clear and relevant, but it could be strengthened by explaining why factors such as education, income, social support, and self-efficacy influence medication literacy. Linking the identified profiles more clearly to real clinical practice, with simple examples of tailored interventions, would improve practical relevance.

**Response:** Special thanks to you for your good comments. In this manuscript, we have added and explained why factors such as education, income, social support, and self-efficacy influence medication literacy. At the same time, we have added some previous simple examples of tailored interventions. Revised portion are marked in blue in the revised manuscript with track changes paper.
5. Limitations Review: The limitations are well presented; however, they could be more explicit regarding how the homogeneous sample and reliance on self-reported data may affect interpretation and generalizability. Framing each limitation as a clear direction for future research would further strengthen this section.

**Response:** Special thanks to you for your good comments. In this manuscript, we have supplemented the limitations, especially the homogeneous sample and reliance on self-reported data affect interpretation and generalizability. At the same time, we have pointed out a clear direction for future research. Revised portion are marked in blue in the revised manuscript with track changes paper.
6. Conclusion Review: The conclusion summarizes the findings well; however, it could be more impactful by emphasizing the practical benefits of identifying medication literacy profiles and by offering one or two clear, actionable recommendations for clinical practice or health policy.

**Response:** Special thanks to you for your good comments. In this manuscript, we have supplemented the practical benefits of identifying medication literacy profiles and offered actionable recommendations for clinical practice. Revised portion are marked in blue in the revised manuscript with track changes paper.

We tried our best to improve the manuscript and made some changes in the manuscript. These changes will not influence the content and framework of the paper. And here we did not list the changes but marked in blue in the revised paper.

We appreciate for Editors/Reviewers’ warm work earnestly, and hope that the correction will meet with approval.

Once again, thank you very much for your comments and suggestions.

Best regards!
Yours sincerely,
              Chongbin Liu

Corresponding author:
Name: Chongbin Liu
E-mail: [liuchongbin1972@126.com](mailto:liuchongbin1972@126.com).
