# Supplementary material for: Profiles of medication literacy and associated factors among young and middle-aged hypertensive patients in China: A latent profile analysis
Source: PLoS One. 2026 Jul 22;21(7):e0352857. doi: 10.1371/journal.pone.0352857 (PMC13390851; doi:10.1371/journal.pone.0352857)
Supplement: Attachment — Submitted filename: Response_to_reviewers_auresp_2.doc [file pone.0352857.s003.doc]

Dear the academic editor and Reviewers,

We appreciate the opportunity to revise our manuscript titled “Profiles of medication literacy and associated factors among young and middle-aged hypertensive patients in China: a latent profile analysis (PONE-D-25-50007R1)” and are grateful for the insightful comments provided by the reviewers. Those comments are all valuable and very helpful for revising and improving our paper, as well as the important guiding significance to our researches. In the following, we have provided detailed responses to each of the reviewers’ comments. Revised portion are marked in blue in the revised manuscript with track changes paper. We have tried our best to make all the revisions clear, and we hope that the revised manuscript meets the requirements for publication. The main corrections in the paper and the responses to the reviewer’s comments are as following:

**Responses to the reviewer’s comments:**

**Reviewer #1:** Thank you for the opportunity to review this manuscript, “Profiles of medication literacy and associated factors among young and middle-aged hypertensive patients in China: a latent profile analysis” The study is a multicenter cross-sectional survey of 544 hypertensive adults aged 18-59 years recruited from two tertiary hospitals (July 15, 2024-November 15, 2024) and applies latent profile analysis (LPA) to identify medication-literacy subgroups, followed by logistic regression to examine associated factors. Below I put forward 3 points to modify the proposal, for your reference：

1. Data Availability statement appears internally inconsistent; this must be corrected to meet PLOS expectations for reproducibility. In the submission materials, “No-some restrictions will apply” is selected, yet the accompanying statement indicates that all data are contained within the manuscript/Supporting Information.
 **Response:** We would like to express our sincere gratitude to the reviewer for your insightful comments and valuable feedback. We have revised the accompanying statement by removing the sentence “all data are contained within the manuscript/Supporting Information.”

2. Methods are not yet sufficiently reproducible for LPA+regression (inputs, missing data, and classification uncertainty). The manuscript indicates use of SPSS 26.0 and Mplus 8.3 and lists fit indices (AIC/BIC/aBIC, entropy, LMR, BLRT), but key implementation details are still missing (e.g., which variables were entered into LPA-18 items vs. domain scores; whether variables were standardized; random starts; missing-data handling).
 **Response:** We would like to express our sincere gratitude to the reviewer for your insightful comments and valuable feedback.

The revised objective now reads: The four domain scores of the Revised Chinese Medication Literacy Scale for Hypertensive Patients (C-MLSHP-R) were used as the input variables for latent profile analysis (Page7, line131-133).

3. Interpretation should be more cautious given cross-sectional design and potential information bias from assisted completion.The study is cross-sectional, sampled from tertiary hospitals, and a subset of respondents had questionnaires read aloud and completed by researchers, which may introduce measurement or social-desirability bias. The study addresses an important applied question and the LPA-derived profiles are promising. However, I recommend making the above 3 changes, primarily to (i) resolve the Data Availability inconsistency, (ii) strengthen reproducibility and appropriately handle classification uncertainty, and (iii) tighten causal language and bias/generalizability discussion in line with the design.

**Response:** We would like to express our sincere gratitude to the reviewer for your insightful comments and valuable feedback.

In discussion part, the revised objective now reads: However, these cross-sectional associations do not establish causal directionality. This design precludes determination of whether these factors precede and influence medication literacy, whether medication literacy shapes these psychosocial attributes, or whether unmeasured confounders account for these relationships. For instance, low medication literacy may lead to suboptimal disease control, subsequently diminishing self-efficacy and amplifying negative illness perceptions. Longitudinal studies are warranted to elucidate these temporal relationships and empirically test the proposed theoretical model (Page14, line286-292).

In limitations part, the revised objective now reads: Second, the cross-sectional study design inherently precludes the establishment of causal relationships between variables and only verifies their associative relationships. Additionally, a small number of illiterate patients had their questionnaires completed by researchers following verbal administration, which may have introduced measurement bias or social desirability bias. Prospective longitudinal studies are warranted in the future to clarify the temporal relationships between variables and adopt more objective evaluation indicators to reduce the impact of bias on study results (Page15, line303-312).

**Reviewer #2:**

1. Introduction: The introduction addresses an important topic; however, it would benefit from stronger conceptual grounding. Key variables such as self-efficacy and illness perception are discussed, yet they are not clearly situated within an established theoretical framework, which somewhat limits the scientific coherence of the study. In addition, the rationale for adopting a latent profile approach is introduced rather abruptly, with limited discussion of the shortcomings of prior variable-centered analyses. A more explicit justification of this methodological choice would strengthen the argument. Finally, the decision to focus on young and middle-aged adults (18-59 years) requires clearer justification, particularly in terms of why this specific age group warrants distinct investigation.

**Response:** We would like to express our sincere gratitude to the reviewer for your insightful comments and valuable feedback.

In the introduction part, the revised objective now reads: This study employs Bandura’s Social Cognitive Theory first articulated in 1986, with particular emphasis on triadic reciprocal determinism and self-efficacy theory. Triadic reciprocal determinism conceptualizes environment, personal cognition, and behavior as interdependent components of a unified system. These elements interact dynamically, with behavior emerging as a product of the continuous interplay between cognitive processes and environmental contexts. Self-efficacy theory posits that individuals’ beliefs in their capabilities exert significant influence on behavioral outcomes. In the present study, medication literacy is operationalized as the behavioral component, social support as the environmental factor, and illness perception as the cognitive element (Page5, line79-86).

In the introduction part, the revised objective now reads: However, this approach overlooks individual differences and group heterogeneity, potentially masking distinct subpopulations with unique medication literacy patterns that require tailored interventions (Page5, line76-78).

In the introduction part, the revised objective now reads: Unlike traditional cluster analysis, LPA offers several advantages: it uses maximum likelihood estimation for greater statistical rigor, provides probabilistic class membership rather than deterministic classification, and offers comprehensive model fit indices to guide class enumeration (Page5, line88-91).

In the introduction part, the revised objective now reads: The focus on young and middle-aged adults (18-59 years) is particularly warranted for several reasons. First, this age group represents a critical window for early intervention, as cardiovascular risk factors established during this period significantly influence later-life outcomes. Second, young and middle-aged hypertensive patients face unique challenges including work-related stress, family responsibilities in China. Third, these hypertensive patients demonstrate particular vulnerability due to three critical factors: heightened health risk perception deficits compared to older adults, inadequate self-management behaviors, and persistent misconceptions equating asymptomatic presentation with therapeutic non-necessity (Page4, line59-66).

2. Methods: While the methodological structure is generally appropriate, several aspects require clarification. The manuscript does not provide a formal power analysis or detailed justification for sample adequacy in the context of latent profile analysis, where class stability and model complexity are important considerations. Furthermore, essential modeling details related to the latent profile procedure—such as estimation methods, model assumptions, and safeguards against local maxima—are not sufficiently described, which limits reproducibility. The regression strategy also requires clearer explanation. The criteria for variable selection and control of potential confounders are not fully articulated, and there is no indication that multicollinearity among predictors was assessed. Finally, the recruitment of participants from only two tertiary hospitals in a single city raises concerns regarding external validity and potential selection bias.
 **Response:** We sincerely thank the reviewer for this thorough evaluation of our methodology. We agree that providing these critical details enhances the transparency, reproducibility, and rigor of our study. We have carefully addressed each of the five methodological concerns raised, as detailed below.

(1) Sample adequacy for Latent Profile Analysis

We appreciate the reviewer’s point regarding the need for a formal justification of sample adequacy. While traditional a priori power analysis for LPA is complex and lacks a single standardized formula, extensive Monte Carlo simulation studies have established robust guidelines for sample size requirements.

Actually, methodological research by Nylund et al. (2007) and Tein et al. (2013) demonstrated that a sample size of approximately 500 is generally sufficient to achieve adequate statistical power (>0.80) to detect the correct number of latent classes, particularly when class separation is distinct. Furthermore, Dziak et al. (2014) noted that high classification accuracy (entropy) significantly reduces the sample size required for stable estimation. Given our sample size of 544 and the excellent class separation observed in our optimal model (entropy=0.927), our study possesses adequate statistical power to support the stability of the identified 3-profile solution.

**References:** Nylund KL, Asparouhov T, Muthén BO. Deciding on the number of classes in latent class analysis and growth mixture modeling: a Monte Carlo simulation study. Struct Equ Modeling. 2007; 14(4): 535-69. http://dx.doi.org/10.1080/10705510701575396.

Tein JY, Coxe S, Cham H. Statistical power to detect the correct number of classes in latent profile analysis. Struct Equ Modeling. 2013; 20(4): 640-57.

http://dx.doi.org/10.1080/10705511.2013.824781.

(2) LPA modeling details

We apologize for the omission of these essential modeling details. In the revised manuscript, we have explicitly described the estimation method, model assumptions, and safeguards against local maxima to ensure full reproducibility.

Specifically, we used the robust Maximum Likelihood Estimator (MLR) to account for potential non-normality in the data. Regarding model assumptions, we specified a model where variances were freely estimated across profiles to capture subgroup-specific variability, while covariances between indicators were constrained to zero within classes (the local independence assumption). To safeguard against local maxima, we employed a rigorous multiple random starts strategy.

(3) Regression strategy and variable selection

We agree that our regression strategy required clearer articulation. We utilized a two-step approach for variable selection. First, bivariate analyses (Chi-square tests and ANOVA) were conducted to identify potential candidate variables associated with medication literacy profiles. Second, variables demonstrating statistical significance (*P*<0.05) in the bivariate analyses, along with theoretically important sociodemographic confounders (e.g., gender), were simultaneously entered into the multivariate multinomial logistic regression model. This approach ensures that the reported associations are adjusted for potential confounding effects.

(4) Multicollinearity assessment

We thank the reviewer for pointing out this critical omission. To ensure the validity of our regression estimates, we have now conducted a formal multicollinearity assessment using the Variance Inflation Factor (VIF). The analysis revealed that the VIF values for all predictors ranged from 1.029 to 3.056. Because all VIF values are well below the conventional threshold of 5 (and the stricter threshold of 3 for most variables), we conclude that multicollinearity does not pose a threat to the stability of our multivariate logistic regression model (Ref. Figures below).

(5) External validity and selection bias

The reviewer rightly points out that recruiting participants from only two tertiary hospitals in a single city limits the external validity of our findings. Patients attending tertiary hospitals may possess different socioeconomic characteristics, health-seeking behaviors, and disease severity compared to those in primary care settings or rural areas. We fully acknowledge this limitation and the potential for selection bias.

We have expanded the limitations section to explicitly address this concern and guide future research.

3. Results: The selection of the three-profile solution would benefit from a more comprehensive justification, particularly given that fit indices continued to improve with additional classes. A clearer discussion of the trade-offs underlying this decision is warranted. In addition, latent class membership was used in subsequent regression analyses without addressing Potential classification uncertainty may influence the accuracy of parameter estimates.The results section places strong emphasis on statistical significance, while offering limited discussion of effect sizes and their clinical implications. Overall, the presentation remains largely descriptive, with limited analytical depth and no robustness or sensitivity analyses to support the stability of the identified profiles.
 **Response:** We would like to express our sincere gratitude to the reviewer for these insightful and constructive comments, which have substantially improved the analytical depth and methodological rigor of our manuscript. We have carefully addressed each of the four concerns raised, as detailed below.

(1) Comprehensive justification for the three-profile solution

We appreciate the reviewer’s observation that information criteria (AIC, BIC, aBIC) continued to decrease with additional classes. This is a well-recognized phenomenon in latent profile analysis: as model complexity increases, fit indices will generally improve because more parameters are being estimated to fit the sample data. However, as Sinha et al.[1] emphasized in their practitioner’s guide, such continuous improvement can lead to overfitting, where the model becomes increasingly unique to the sample and less generalizable to the population. Therefore, model selection in LPA must balance statistical fit with the principle of parsimony, classification quality, and substantive interpretability [1-2].

We selected the 3-profile model based on the following comprehensive evaluation of trade-offs:

a. Statistical fit indices and likelihood ratio tests. Although the 4-profile and 5-profile models yielded lower AIC and BIC values, the Lo-Mendell-Rubin (LMR) adjusted likelihood ratio test for the 4-profile model was not statistically significant (*P*=0.108), indicating that adding a fourth class did not significantly improve model fit over the 3-profile solution. In contrast, the LMR test for the 3-profile model was highly significant (*P*<0.001), confirming that three classes fit the data significantly better than two classes.

b. Smallest class size. The 5-profile model produced a class containing only 0.9% of the sample (approximately 5 individuals), which falls well below the commonly recommended threshold of 5% for meaningful interpretation and model stability [1,3]. Such extremely small classes are often indicative of overfitting or data artifacts rather than substantively meaningful subgroups. The 4-profile model also contained a relatively small class (5.3%), raising similar concerns about stability and generalizability.

c. Classification quality. The 3-profile model demonstrated excellent classification accuracy, with an entropy value of 0.927 (well above the 0.80 threshold commonly recommended for adequate class separation) and high average latent class probabilities of 97.2%, 96.7%, and 97.9% for the three profiles, respectively. These values indicate that individuals were assigned to their most likely class with high confidence.

d. Theoretical coherence and clinical interpretability. The three identified profiles (low, moderate, and high medication literacy) align with a theoretically meaningful gradient that is directly actionable for clinical practice. Also, this tripartite classification is consistent with Social Cognitive Theory and provides a clear framework for developing stratified interventions tailored to each subgroup’s specific needs.

We have expanded the discussion of these trade-offs in the revised manuscript.

**References:**

[1] Sinha P, Calfee CS, Delucchi KL. Practitioner’s Guide to Latent Class Analysis: Methodological Considerations and Common Pitfalls. Crit Care Med. 2021; 49(1): e63-e79. doi:10.1097/CCM.0000000000004710. PMID: 33165028.

[2] Weller BE, Bowen NK, Faubert SJ. Latent class analysis: a guide to best practice. J Black Psychol. 2020; 46(4): 287-311. doi:10.1177/0095798420930932.

[3] Nylund KL, Asparouhov T, Muthén BO. Deciding on the number of classes in latent class analysis and growth mixture modeling: a Monte Carlo simulation study. Struct Equ Modeling. 2007; 14(4): 535-69. doi:10.1080/10705510701575396.

(2) Classification uncertainty in subsequent regression analyses

We thank the reviewer for raising this important methodological concern. When latent class membership is treated as a known categorical variable in subsequent regression analyses (the "classify-then-analyze" approach), classification error can attenuate parameter estimates and introduce bias [4,5]. We acknowledge this limitation and have addressed it in the revised manuscript.

However, we note that the potential bias in our study is substantially mitigated by the high classification accuracy of our 3-profile model. The entropy value of 0.927 and the average posterior probabilities exceeding 96% for all three profiles indicate that the vast majority of participants were classified with high certainty. Methodological research has demonstrated that when entropy is high (typically>0.80) and posterior probabilities approach 1.0, the information loss introduced by modal class assignment is minimal, and the resulting parameter estimates closely approximate those obtained from more sophisticated bias-adjusted methods such as the BCH approach or the three-step ML method [4,6,7].

We have added the following clarifications to the revised manuscript.

**References:**

[4] Vermunt JK. Latent class modeling with covariates: two improved three-step approaches. Polit Anal. 2010; 18(4):450-69. doi:10.1093/pan/mpq025.

[5] Asparouhov T, Muthén B. Auxiliary variables in mixture modeling: using the BCH method in Mplus to estimate a distal outcome model and an arbitrary secondary model. Mplus Web Notes. 2021; No. 21, Version 11.

[6] Asparouhov T, Muthén B. Auxiliary variables in mixture modeling: three-step approaches using Mplus. Struct Equ Modeling. 2014; 21(3):329-41. doi:10.1080/10705511.2014.915181.

[7] Bray BC, Lanza ST, Tan X. Eliminating bias in classify-analyze approaches for latent class analysis. Struct Equ Modeling. 2015; 22(1):1-11. doi:10.1080/10705511.2014.935265.

(3) Effect sizes and clinical implications

We agree with the reviewer that our previous presentation placed excessive emphasis on statistical significance (*P*-values) while offering limited discussion of effect sizes and their clinical meaning. We have substantially revised the Results and Discussion sections to foreground effect sizes (odds ratios and their 95% confidence intervals) and to explicitly discuss their clinical implications.

(4) Robustness and sensitivity analyses

We thank the reviewer for emphasizing the importance of robustness checks to support the stability of the identified profiles. In response, we have conducted additional analyses and reported them in the revised manuscript.

a. Random starts and convergence. To ensure that our LPA models were not trapped in local maxima, we used 500 random starts with 100 final stage optimizations during the estimation process. The best log-likelihood value was successfully replicated across multiple random starts for all models tested (1-through 5-profile solutions), confirming that the solutions represent global rather than local maxima [1,8].

b. Split-half cross-validation. To evaluate the stability of the 3-profile solution, we conducted a random split-half validation. The full sample (n=544) was randomly divided into two subsamples (n₁=272, n₂=272), and LPA was performed independently on each subsample. In both subsamples, the 3-profile model emerged as the optimal solution based on the same criteria applied to the full sample (non-significant LMR for the 4-profile model; smallest class proportion below 5% for the 5-profile model). The profile proportions and mean domain scores in both subsamples were consistent with those observed in the full-sample analysis, supporting the stability and replicability of the identified profiles.We have added a new subsection "3.4 Sensitivity Analysis" to the Results section and updated the Methods section to detail these validation procedures.

We believe these revisions comprehensively address the reviewer’s concerns by providing (1) a thorough justification for the three-profile solution with explicit discussion of trade-offs, (2) acknowledgment and mitigation of classification uncertainty, (3) enhanced reporting of effect sizes and their clinical implications, and (4) robustness and sensitivity analyses supporting the stability of the identified profiles.

**References:**

[8] Finch WH, Bronk KC. Conducting confirmatory latent class analysis using Mplus. Struct Equ Modeling. 2011; 18(1):132-51. doi:10.1080/10705511.2011.532732.

4. Discussion: The discussion summarizes the findings effectively but would benefit from deeper analytical engagement. Much of the section reiterates results rather than exploring underlying mechanisms or situating the findings within broader theoretical perspectives. Given the cross-sectional design, some interpretations and practice-oriented recommendations should be framed more cautiously to avoid implying causal relationships. A more explicit reflection on methodological limitations, particularly those related to the latent profile approach, would also strengthen this section.
 **Response:** We would like to express our sincere gratitude to the reviewer for your insightful comments and valuable feedback.

In the discussion part, the revised objective now reads: Grounded in Social Cognitive Theory, the identified medication literacy profiles indicate that medication literacy extends beyond knowledge acquisition. It reflects a dynamic interplay among cognitive factors (self-efficacy, illness perception), environmental influences (social support), and behavioral determinants. These profile-specific characteristics suggest tailored intervention strategies: low-literacy patients may benefit from foundational knowledge and skills training, moderate-literacy patients require reinforcement of positive attitudes and behaviors, and high-literacy patients could serve as peer educators. This theoretically grounded stratification transcends descriptive classification. It elucidates specific mechanistic pathways to inform tailored interventions (Page11-12, line225-232).

In the discussion part, the revised objective now reads: The observed associations among social support, self-efficacy, illness perception, and medication literacy profiles are consistent with Social Cognitive Theory. However, these cross-sectional associations do not establish causal directionality. This design precludes determination of whether these factors precede and influence medication literacy, whether medication literacy shapes these psychosocial attributes, or whether unmeasured confounders account for these relationships. For instance, low medication literacy may lead to suboptimal disease control, subsequently diminishing self-efficacy and amplifying negative illness perceptions. Longitudinal studies are warranted to elucidate these temporal relationships and empirically test the proposed theoretical model (Page14, line284-292).

4. Limitations and Conclusion: Although general limitations are acknowledged, there is limited discussion of issues specific to the modeling approach, such as model selection uncertainty or classification error. Moreover, the connection between stated limitations and the strength of the conclusions could be more clearly articulated.
   The conclusion largely restates the main findings without clearly highlighting the study’s theoretical or methodological contribution. Practical recommendations appear somewhat stronger than what can be supported by an observational design and would benefit from more cautious framing.
    **Response:** We would like to express our sincere gratitude to the reviewer for your insightful comments and valuable feedback.

In the limitations part, the revised objective now reads: Second, the cross-sectional study design inherently precludes the establishment of causal relationships between variables and only verifies their associative relationships. Additionally, while the 3-profile model was identified as optimal, and the high entropy and posterior probabilities of our model mitigate classification error, the classify-then-analyze approach used in our regression models may still introduce some degree of bias in parameter estimates. Third, a small number of illiterate patients had their questionnaires completed by researchers following verbal administration, which may have introduced measurement bias or social desirability bias. Prospective longitudinal studies are warranted in the future to clarify the temporal relationships between variables and adopt more objective evaluation indicators to reduce the impact of bias on study results (Page15, line303-312).

In the conclusion part, the revised objective now reads: These findings extend Social Cognitive Theory to medication literacy research by demonstrating how personal, environmental, and behavioral factors differentiate patient subgroups, contributing to the theoretical understanding of health literacy development in chronic disease contexts (Page16, line320-323).

In the conclusion part, the revised objective now reads: However, given the observational design and methodological constraints discussed, these recommendations should be regarded as preliminary and hypothesis-generating rather than evidence-based clinical guidelines (Page16, line325-328).

6. Abstract: The abstract would benefit from a more balanced structure. Clinical implications appear somewhat overstated given the study design, and key limitations are not mentioned. Additionally, methodological details receive relatively greater emphasis than interpretive insights, which could be better prioritized in a concise summary.

**Response:** We would like to express our sincere gratitude to the reviewer for your insightful comments and valuable feedback.

In abstract part. the revised objective now reads: More specifically, lower education and income predicted membership in the low literacy profile, while higher social support, self-efficacy, and positive illness perception characterized the high literacy group. These results support stratified interventions: foundational education for low-literacy patients, behavioral reinforcement for moderate-literacy patients, and enhanced psychosocial support for disadvantaged groups. Causal interpretations are limited by the cross-sectional design, necessitating future longitudinal validation. (Page3, line46-52).

We tried our best to improve the manuscript and made some changes in the manuscript. These changes will not influence the content and framework of the paper. And here we did not list the changes but marked in blue in the revised paper.

We appreciate for Editors/Reviewers’ warm work earnestly, and hope that the correction will meet with approval.

Once again, thank you very much for your comments and suggestions.

Best regards!
Yours sincerely,
              Chongbin Liu

Corresponding author:
Name: Chongbin Liu
E-mail: [liuchongbin1972@126.com](mailto:liuchongbin1972@126.com).
