# Supplementary material for: Profiles of medication literacy and associated factors among young and middle-aged hypertensive patients in China: A latent profile analysis
Source: PLoS One. 2026 Jul 22;21(7):e0352857. doi: 10.1371/journal.pone.0352857 (PMC13390851; doi:10.1371/journal.pone.0352857)
Supplement: Attachment — Submitted filename: Response_to_reviewers_auresp_3.doc [file pone.0352857.s004.doc]

Dear the academic editor and Reviewers,

We appreciate the opportunity to revise our manuscript titled “Profiles of medication literacy and associated factors among young and middle-aged hypertensive patients in China: a latent profile analysis (PONE-D-25-50007R2)” and are grateful for the insightful comments provided by the reviewers. Those comments are all valuable and very helpful for revising and improving our paper, as well as the important guiding significance to our researches. In the following, we have provided detailed responses to each of the reviewers’ comments. Revised portion are marked in blue in the revised manuscript with track changes paper. We have tried our best to make all the revisions clear, and we hope that the revised manuscript meets the requirements for publication. The main corrections in the paper and the responses to the reviewer’s comments are as following:

**Responses to the reviewer’s comments:**

**Reviewer #1:** The study revolves around the heterogeneity of drug literacy in young and middle-aged patients with hypertension, adopts a cross-sectional design, consecutively enrolls 544 patients from two tertiary hospitals, and conducts a potential profile analysis using the four dimensions of C-MLSHP-R, and analyzes the relationship between drug literacy and risk factors, and further test the related factors with multinomial Logistic regression. The authors identified three subtypes of drug literacy: low, moderate, and high, and reported that education level, family income, social support, self-efficacy, and disease perception were significantly associated with subtype attribution. According to PLOS ONE’s criteria of “Only judging scientific rigor”, the advantages of this paper are clear research questions, complete ethical approval and informed consent, and more complete modeling details than common cross-sectional studies, the random starting point, VIF and split-half sensitivity analysis are supplemented, which shows that the authors have made efforts to improve the reproducibility and stability of the results. However, there are still some key issues that affect the judgment of publication:
 1. Data availability statements are still not enough to sati PLOSONE’s reproducibility requirements. Data availability information is still unclear from the current manuscript package, and the authors themselves admit to previous inconsistencies during the round-trip review.
Recommendation: Please include additional subjects in flow charts or tables and at least report: (a) specific decision rules for 29 invalid questionnaires; (b) the proportion missing for each core variable/scale; (C) LPA versus treatment of missingness used in regression; (d) in the absence of missing data, state explicitly “No missing data for variables entered into LPA/regression”.
 **Response:** We would like to express our sincere gratitude to the reviewer for your insightful comments and valuable feedback. We have supplemented additional subjects in results portion in this text.

2. The external validity is limited, while the practical recommendations are still slightly strong. The sample was collected from two tertiary hospitals in the same city in China, and a small number of illiterate patients were filled in by the offspring, which may introduce selection bias and information bias. Although the authors mention this in their limitations, the conclusion “Provides a theoretical basis for the development of intervention programs for caregivers” and “Should be individualized,” which is slightly forward in the hierarchy of evidence.

**Response:** We would like to express our sincere gratitude to the reviewer for your insightful comments and valuable feedback. We have corrected the language in the conclusion, which is slightly forward in the hierarchy of evidence.

3. On the basis of univariate significant variables and theoretically relevant confounders, multinomial Logistic regression was entered; Table 4 also encodes variables such as age, place of residence, occupational status, type of work, and blood pressure monitoring. However, Table 5 ultimately reported only five categories of variables: education, income, PSSS, GSES, and BIPQ, complete adjustment for age, place of residence, occupation, type of work, blood pressure monitoring, or theoretical confounders such as sex was not seen.

**Response:** Thank you for this important and constructive comment. We agree that the covariates included in the multinomial logistic regression model should be reported more transparently. In the revised manuscript, we re-ran the multinomial logistic regression using a fully adjusted model. In addition to education level, per capita monthly household income, PSSS, GSES, and BIPQ, we included theoretically relevant sociodemographic and disease-management covariates, including age, sex, place of residence, occupational/work-related status, and blood pressure monitoring.

During model re-specification, we found that occupational status and type of work were highly overlapping in the dataset, particularly because retired participants were simultaneously coded as “retired/neither” for type of work. To avoid multicollinearity and unstable parameter estimates, we handled these two variables as occupational/work-related status in the adjusted model. All retained covariates were kept in the model regardless of statistical significance. We have revised the Statistical analysis section to clarify the model-building strategy, added sex/gender coding to the variable assignment table, and expanded Table 5 to present the fully adjusted multinomial logistic regression results, including both statistically significant and non-significant covariates. The Results section and table footnotes have also been revised accordingly.

4. Language and grammar errors are still more, it is recommended to Polish professional English.
 **Response:** We would like to express our sincere gratitude to the reviewer for your insightful comments and valuable feedback. We have tried our best to improve the language and grammar errors in this manuscript and made some changes in the manuscript.

5. There are spelling and typographical errors in the table. table1 and table 5.
 **Response:** We would like to express our sincere gratitude to the reviewer for your insightful comments and valuable feedback. We have checked and corrected the spelling and typographical errors in the table1 and table 5.
 6. It is suggested that the scale reliability index of the current sample should be supplemented in the text.
 **Response:** We would like to express our sincere gratitude to the reviewer for your insightful comments and valuable feedback. The reliability of the scales was assessed using Cronbach’s α coefficient in this study. We have supplemented in the text.
 7. References and format details should be unified.
 **Response:** We would like to express our sincere gratitude to the reviewer for your insightful comments and valuable feedback. We have checked and corrected references and format details in the text.

8. In the results section, it is suggested to reduce“Statistically significant” repetitive statements and increase model interpretation.

**Response:** We would like to express our sincere gratitude to the reviewer for your insightful comments and valuable feedback. In the results section, we have reduced the “Statistically significant” repetitive statements.

**Reviewer #2:** The manuscript has improved and most comments have been addressed well. Only one minor point: Please briefly clarify the sample size justification for the latent profile analysis in the Methods section. Overall, the manuscript is suitable for publication after this minor revision.

**Response:** We would like to express our sincere gratitude to the reviewer for your insightful comments and valuable feedback. We have clarified the sample size justification for the latent profile analysis in the Methods section.

We tried our best to improve the manuscript and made some changes in the manuscript. These changes will not influence the content and framework of the paper. And here we did not list the changes but marked in blue in the revised paper.

We appreciate for Editors/Reviewers’ warm work earnestly, and hope that the correction will meet with approval.

Once again, thank you very much for your comments and suggestions.

Best regards!
Yours sincerely,
              Rui Wang
