# Supplementary material for: Profiles of medication literacy and associated factors among young and middle-aged hypertensive patients in China: A latent profile analysis
Source: PLoS One. 2026 Jul 22;21(7):e0352857. doi: 10.1371/journal.pone.0352857 (PMC13390851; doi:10.1371/journal.pone.0352857)
Supplement: Attachment — Submitted filename: Response_to_reviewers_auresp_4.doc [file pone.0352857.s005.doc]

Dear the academic editor and Reviewers,

We appreciate the opportunity to revise our manuscript titled “Profiles of medication literacy and associated factors among young and middle-aged hypertensive patients in China: a latent profile analysis (PONE-D-25-50007R3)” and are grateful for the insightful comments provided by the reviewers. In the following, we have provided detailed responses to each of the reviewers’ comments. Revised portion are marked in blue in the revised manuscript with track changes paper. We have tried our best to make all the revisions clear, and we hope that the revised manuscript meets the requirements for publication. The main corrections in the paper and the responses to the reviewer’s comments are as following:

**Responses to the reviewer’s comments:**

**Reviewer #1:**

1. Use “moderate medication literacy” consistently instead of alternating between “medium” and “moderate.” Replace full-width symbols such as “＜” with standard mathematical symbols.

**Response:** We would like to express our sincere gratitude to the reviewer for your insightful comments and valuable feedback. We have corrected these imperfection in the revised manuscript with track changes.

2. Use χ² or “Chi-square” instead of “X2.”

**Response:** We would like to express our sincere gratitude to the reviewer for your insightful comments and valuable feedback. We have also corrected these imperfection in the revised manuscript with track changes.

3. Explain the scoring range and direction for each scale and each C-MLSHP-R domain.

**Response:** We would like to express our sincere gratitude to the reviewer for your insightful comments and valuable feedback. We have supplemented the scoring range and direction for each scale and each C-MLSHP-R domain in the revised manuscript with track changes.
 4. Please check all DOI formatting; at least one DOI appears to contain an extra space. Avoid speculative statements in the Discussion unless clearly marked as possible explanations. For example, “Maybe people with high incomes…” should be rewritten in formal academic language.

**Response:** We would like to express our sincere gratitude to the reviewer for your insightful comments and valuable feedback. We have also corrected DOI formatting and speculative statements in the revised manuscript with track changes.
 5. The funding statement should include whether the funder had any role in study design, data collection and analysis, decision to publish, or manuscript preparation.
 **Response:** We would like to express our sincere gratitude to the reviewer for your insightful comments and valuable feedback. We have supplemented the funding statement in the title page in the revised manuscript with track changes.

6. Please avoid overstating external validity. Since the sample was recruited from two tertiary hospitals in one city, the study should be described as a two-hospital, single-city, hospital-based cross-sectional study.
 **Response:** We would like to express our sincere gratitude to the reviewer for your insightful comments and valuable feedback. We have also corrected these imperfection in the revised manuscript with track changes.

**Reviewer #2:** Thank you for the authors’ efforts in revising the manuscript. The paper has clearly improved, and most of the previous comments have been addressed. I only have a few minor suggestions that may help improve the clarity and transparency of the manuscript before final acceptance.
 1. Sample size justification for LPA
 The sample size is mentioned as adequate, but it would be helpful if the authors briefly clarify why 544 participants are sufficient for latent profile analysis.
 **Response:** We would like to express our sincere gratitude to the reviewer for your insightful comments and valuable feedback. Actually, in our paper, the references (14, 19 and 20) are all shown that “best-practice and review results concluded that a minimum sample size of about 500 should lead to enough accuracy in identifying a correct number of latent profiles”.

2. Data availability

Please provide a clearer Data Availability Statement, including the repository name, DOI, accession number, or link. If the data cannot be shared openly, the reason should be briefly explained.

**Response:** We would like to express our sincere gratitude to the reviewer for your insightful comments and valuable feedback. We have supplemented the data availability statement in the title page in the revised manuscript with track changes.

3. Invalid questionnaires and missing data

The explanation of the 29 excluded questionnaires is clear, but it may be better placed in the Methods section or shown in a simple participant flow chart.
 **Response:** We would like to express our sincere gratitude to the reviewer for your insightful comments and valuable feedback. The explanation of the 29 excluded questionnaires have placed in the Methods section in the revised manuscript with track changes.

4. Table 5 and unstable ORs

Some odds ratios in Table 5 appear unstable because of zero cases in certain categories. The authors may either address this statistically, for example by merging sparse categories or using penalized multinomial logistic regression, or clearly mention that these estimates should be interpreted with caution.
 **Response:** We would like to express our sincere gratitude to the reviewer for your insightful comments and valuable feedback. As the reviewer noted that some OR values in Table 5 exhibit wide confidence intervals. This phenomenon is attributable to data sparsity in the high medication literacy profile (Profile 3, n = 79). This sparse data situation leads to quasi-complete separation in logistic regression, where the maximum likelihood estimator becomes unstable, producing inflated OR values and extremely wide confidence intervals. We acknowledge this limitation and have taken the following steps in the revised manuscript:

a) Added cautionary interpretation in Results: It should be noted that some OR estimates (e.g., education: junior middle school and below) exhibited wide confidence intervals due to sparse cell counts in the high medication literacy profile (n = 79). These estimates should be interpreted with caution, and the direction of association rather than the precise magnitude is more informative in these cases."

b) Added acknowledgment in Limitations: “Fourth, the relatively small sample size of the high medication literacy profile (n = 79) limited the number of covariates that could be reliably included in the multinomial logistic regression model. The sparse data in this subgroup led to wide confidence intervals for some OR estimates, particularly for education and income categories. Future studies with larger sample sizes are needed to provide more precise estimates and to allow for comprehensive covariate adjustment including demographic confounders such as age, sex, and place of residence”.

5. Discussion and practical recommendations

The intervention-related recommendations are useful, but they should be expressed more cautiously. Please clarify that these recommendations are preliminary and hypothesis-generating, rather than clinical guidelines.
 **Response:** We would like to express our sincere gratitude to the reviewer for your insightful comments and valuable feedback. We have supplemented the statement in the revised manuscript with track changes.

6. Generalizability

Please clarify that although the sample size may be adequate for LPA, the recruitment from two hospitals in one city may limit the generalizability of the findings.

**Response:** We would like to express our sincere gratitude to the reviewer for your insightful comments and valuable feedback. We have supplemented the statement in the manuscript.

Once again, thank you very much for your comments and suggestions.

Best regards!
Yours sincerely,
              Rui Wang
