# Supplementary material for: Profiles of medication literacy and associated factors among young and middle-aged hypertensive patients in China: A latent profile analysis
Source: PLoS One. 2026 Jul 22;21(7):e0352857. doi: 10.1371/journal.pone.0352857 (PMC13390851; doi:10.1371/journal.pone.0352857)
Supplement: Peer review history

# Peer review: Decision Letter 0

**DOI:** 10.1371/journal.pone.0352857.r001

**Authors:** Wejdan Shahin

29 Dec 2025

PONE-D-25-50007

Profiles of medication literacy and its predictors among young and middle-aged hypertensive patients in China: a latent profile analysis

PLOS One

Dear Dr. Wang,

Thank you for submitting your manuscript to PLOS ONE. After careful consideration, we feel that it has merit but does not fully meet PLOS ONE’s publication criteria as it currently stands. Therefore, we invite you to submit a revised version of the manuscript that addresses the points raised during the review process.

==============================

The manuscript presents a timely and methodologically sound application of latent profile analysis to explore heterogeneity in medication literacy among young and middle-aged patients with hypertension. The person-centred analytical approach is a clear strength and offers added value beyond traditional variable-centred methods. However, in its current form, the manuscript requires substantial refinement to ensure interpretive caution, conceptual clarity, and stronger translational relevance.

The primary areas requiring revision relate to how the study scope and findings are framed and interpreted. The manuscript would benefit from clearer definition and justification of the target population early in the Introduction, along with a more explicitly articulated research focus that guides the reader through the study rationale. While the analytical methods are appropriate, several sections of the Results and Discussion employ language that may be interpreted as implying causality, despite the cross-sectional design. A more cautious interpretive stance is needed, particularly when describing associations between medication literacy profiles and sociodemographic or psychosocial variables.

Greater transparency is also required regarding the generalisability of the findings. The exclusive recruitment from tertiary hospitals in an economically developed region, combined with the focus on a single age group and reliance on self-reported measures, constrains the extent to which the results can be extrapolated to broader populations. These issues should be addressed more explicitly in the Limitations section and reframed as opportunities for future research, including longitudinal designs and recruitment across diverse geographic and healthcare contexts.

==============================

Please submit your revised manuscript by Feb 12 2026 11:59PM. If you will need more time than this to complete your revisions, please reply to this message or contact the journal office at plosone@plos.org. When you're ready to submit your revision, log on to https://www.editorialmanager.com/pone/ and select the 'Submissions Needing Revision' folder to locate your manuscript file.

Please include the following items when submitting your revised manuscript:

- A letter that responds to each point raised by the academic editor and reviewer(s). You should upload this letter as a separate file labeled 'Response to Reviewers'.
- A marked-up copy of your manuscript that highlights changes made to the original version. You should upload this as a separate file labeled 'Revised Manuscript with Track Changes'.
- An unmarked version of your revised paper without tracked changes. You should upload this as a separate file labeled 'Manuscript'.

If you would like to make changes to your financial disclosure, please include your updated statement in your cover letter. Guidelines for resubmitting your figure files are available below the reviewer comments at the end of this letter.

If applicable, we recommend that you deposit your laboratory protocols in protocols.io to enhance the reproducibility of your results. Protocols.io assigns your protocol its own identifier (DOI) so that it can be cited independently in the future. For instructions see: https://journals.plos.org/plosone/s/submission-guidelines#loc-laboratory-protocols. Additionally, PLOS ONE offers an option for publishing peer-reviewed Lab Protocol articles, which describe protocols hosted on protocols.io. Read more information on sharing protocols at https://plos.org/protocols?utm_medium=editorial-email&utm_source=authorletters&utm_campaign=protocols.

We look forward to receiving your revised manuscript.

Kind regards,

Wejdan Shahin

Academic Editor

PLOS One

Journal requirements:

When submitting your revision, we need you to address these additional requirements.

1. Please ensure that your manuscript meets PLOS ONE's style requirements, including those for file naming. The PLOS ONE style templates can be found at

https://journals.plos.org/plosone/s/file?id=wjVg/PLOSOne_formatting_sample_main_body.pdf and

https://journals.plos.org/plosone/s/file?id=ba62/PLOSOne_formatting_sample_title_authors_affiliations.pdf

2. We noticed you have some minor occurrence of overlapping text with the following previous publication(s), which needs to be addressed:

https://www.frontiersin.org/journals/public-health/articles/10.3389/fpubh.2022.987526/full

In your revision ensure you cite all your sources (including your own works), and quote or rephrase any duplicated text outside the methods section. Further consideration is dependent on these concerns being addressed.

3. We note that the grant information you provided in the ‘Funding Information’ and ‘Financial Disclosure’ sections do not match.

When you resubmit, please ensure that you provide the correct grant numbers for the awards you received for your study in the ‘Funding Information’ section.

4. Thank you for stating the following financial disclosure:

“This study was funded by Natural Science Foundation of Huzhou city, Zhejiang province, China (2022YZ44).”

Please state what role the funders took in the study.  If the funders had no role, please state: "The funders had no role in study design, data collection and analysis, decision to publish, or preparation of the manuscript."

If this statement is not correct you must amend it as needed.

Please include this amended Role of Funder statement in your cover letter; we will change the online submission form on your behalf.

5. Thank you for stating the following in the Funding Section of your manuscript:

“This study was funded by Natural Science Foundation of Huzhou city, Zhejiang province, China (2022YZ44).”

We note that you have provided funding information that is currently declared in your Funding Statement. However, funding information should not appear in the Acknowledgments section or other areas of your manuscript. We will only publish funding information present in the Funding Statement section of the online submission form.

Please remove any funding-related text from the manuscript and let us know how you would like to update your Funding Statement. Currently, your Funding Statement reads as follows:

“This study was funded by Natural Science Foundation of Huzhou city, Zhejiang province, China (2022YZ44).”

Please include your amended statements within your cover letter; we will change the online submission form on your behalf.

6. When completing the data availability statement of the submission form, you indicated that you will make your data available on acceptance. We strongly recommend all authors decide on a data sharing plan before acceptance, as the process can be lengthy and hold up publication timelines. Please note that, though access restrictions are acceptable now, your entire data will need to be made freely accessible if your manuscript is accepted for publication. This policy applies to all data except where public deposition would breach compliance with the protocol approved by your research ethics board. If you are unable to adhere to our open data policy, please kindly revise your statement to explain your reasoning and we will seek the editor's input on an exemption. Please be assured that, once you have provided your new statement, the assessment of your exemption will not hold up the peer review process.

7. Your ethics statement should only appear in the Methods section of your manuscript. If your ethics statement is written in any section besides the Methods, please delete it from any other section.

If the reviewer comments include a recommendation to cite specific previously published works, please review and evaluate these publications to determine whether they are relevant and should be cited. There is no requirement to cite these works unless the editor has indicated otherwise.

[Note: HTML markup is below. Please do not edit.]

Reviewers' comments:

Reviewer's Responses to Questions

**Comments to the Author**

1. Is the manuscript technically sound, and do the data support the conclusions?

The manuscript must describe a technically sound piece of scientific research with data that supports the conclusions. Experiments must have been conducted rigorously, with appropriate controls, replication, and sample sizes. The conclusions must be drawn appropriately based on the data presented.

Reviewer #1: Yes

Reviewer #2: Yes

**********

2. Has the statistical analysis been performed appropriately and rigorously?

Reviewer #1: Yes

Reviewer #2: Yes

**********

3. Have the authors made all data underlying the findings in their manuscript fully available?

The PLOS Data policy requires authors to make all data underlying the findings described in their manuscript fully available without restriction, with rare exception (please refer to the Data Availability Statement in the manuscript PDF file). The data should be provided as part of the manuscript or its supporting information, or deposited to a public repository. For example, in addition to summary statistics, the data points behind means, medians and variance measures should be available. If there are restrictions on publicly sharing data—e.g. participant privacy or use of data from a third party—those must be specified.

Reviewer #1: Yes

Reviewer #2: Yes

**********

4. Is the manuscript presented in an intelligible fashion and written in standard English?

PLOS ONE does not copyedit accepted manuscripts, so the language in submitted articles must be clear, correct, and unambiguous. Any typographical or grammatical errors should be corrected at revision, so please note any specific errors here.

Reviewer #1: Yes

Reviewer #2: Yes

**********

5. Review Comments to the Author

Please use the space provided to explain your answers to the questions above. You may also include additional comments for the author, including concerns about dual publication, research ethics, or publication ethics. (Please upload your review as an attachment if it exceeds 20,000 characters)

Reviewer #1: Much appreciated for giving me the opportunity for this review, this study innovatively uses latent class analysis to explore group heterogeneity in drug literacy, going beyond the limitations of traditional variable-centric analysis. This“Human-centered” approach can more truly reflect the diversity and complexity of the patient population, and has important methodological value for developing precise and personalized clinical intervention strategies. But there are some areas for improvement, which should be more fully addressed in the“Limitations” of the discussion section, it is pointed out that the conclusions of this study are mainly applicable to patients in tertiary hospitals in economically developed areas similar to Zhejiang province in China. In conclusion or future research directions, it is suggested that follow-up studies should recruit samples from more diverse areas (such as Midwestern Sectional Figure Skating Championships, urban and rural communities) to verify the generalizability of the findings of this study. More cautious language should be used when discussing partial interpretation of the results, describing“Influencing factors” or“Predictors” as“Relevant factors” or“Association factors”, avoid words that imply causality. The overall language of the manuscript is fluent, but there are minor grammatical problems or repetitions in individual sentences. It is recommended that the final version be thoroughly polished in English. For example, the abstract and the introduction are somewhat repetitive and could be further refined.

Reviewer #2: Dear Authors,

Please find my brief and constructive comments below.

Introduction Review

The introduction is clear; however, defining young and middle-aged patients at the outset would help readers. In addition, briefly clarifying the research gap and stating a clear research question would make the study’s focus easier to follow.

Methodology Review

The study is valuable and provides useful findings; however, a few points could be clarified to improve transparency. As the study uses a cross-sectional design, the results reflect associations only and do not allow for causal interpretations. Moreover, reliance on self-reported questionnaires means that some responses may not fully reflect patients’ actual experiences or behaviors. Focusing on one age group and a single geographic region may also limit the generalizability of the findings. Clearly acknowledging these points in the limitations section would strengthen the manuscript.

Results Review

The study is well conducted; however, the choice of the number of profiles involves some degree of judgment. Therefore, the findings should be interpreted with caution, particularly given the cross-sectional design and limited sample. Acknowledging the potential impact of self-reported data on result accuracy would further strengthen the results section.

Discussion Review

The discussion is clear and relevant, but it could be strengthened by explaining why factors such as education, income, social support, and self-efficacy influence medication literacy. Linking the identified profiles more clearly to real clinical practice, with simple examples of tailored interventions, would improve practical relevance.

Limitations Review

The limitations are well presented; however, they could be more explicit regarding how the homogeneous sample and reliance on self-reported data may affect interpretation and generalizability. Framing each limitation as a clear direction for future research would further strengthen this section.

Conclusion Review

The conclusion summarizes the findings well; however, it could be more impactful by emphasizing the practical benefits of identifying medication literacy profiles and by offering one or two clear, actionable recommendations for clinical practice or health policy.

**********

6. PLOS authors have the option to publish the peer review history of their article (what does this mean?). If published, this will include your full peer review and any attached files.

If you choose “no”, your identity will remain anonymous but your review may still be made public.

**Do you want your identity to be public for this peer review?**  For information about this choice, including consent withdrawal, please see our Privacy Policy.

Reviewer #1: No

Reviewer #2: No

**********

[NOTE: If reviewer comments were submitted as an attachment file, they will be attached to this email and accessible via the submission site. Please log into your account, locate the manuscript record, and check for the action link "View Attachments". If this link does not appear, there are no attachment files.]

To ensure your figures meet our technical requirements, please review our figure guidelines: https://journals.plos.org/plosone/s/figures

You may also use PLOS’s free figure tool, NAAS, to help you prepare publication quality figures: https://journals.plos.org/plosone/s/figures#loc-tools-for-figure-preparation.

NAAS will assess whether your figures meet our technical requirements by comparing each figure against our figure specifications.

# Author comment: Author response to Decision Letter 1

**DOI:** 10.1371/journal.pone.0352857.r002

14 Jan 2026

Dear editors,

We would like to submit the revised manuscript entitled “Profiles of medication literacy and associated factors among young and middle-aged hypertensive patients in China: a latent profile analysis (PONE-D-25-50007)”, which we wish to be considered for publication in “PLOS ONE”.

We also described all authors listed have contributed to the work, all authors have read, approved and agreed to submit the revised manuscript to “PLOS ONE”, no part of the work has been published before.

This study was funded by Natural Science Foundation of Huzhou city, Zhejiang province, China (2022YZ44). The funder role is writing the original draft and investigation.

We have considered all issues mentioned in the academic editor and Reviewers’ comments carefully and have made correction which we hope meet with approval. Revised portion are marked in blue in the revised manuscript with track changes paper.

Thank you and best regards,

yours sincerely,

Chongbin Liu

Corresponding author: School of Medicine, Huzhou University, Huzhou, People’s Republic of China, 313000

E-mail address: liuchongbin1972@126.com.

Attachment Submitted filename: Response to reviewers.doc

# Peer review: Decision Letter 1

**DOI:** 10.1371/journal.pone.0352857.r003

**Authors:** Wejdan Shahin, Wejdan Shahin

17 Feb 2026

PONE-D-25-50007R1

Profiles of medication literacy and associated factors among young and middle-aged hypertensive patients in China: a latent profile analysis

PLOS One

Dear Dr. wang,

Thank you for submitting your manuscript to PLOS ONE. After careful consideration, we feel that it has merit but does not fully meet PLOS ONE’s publication criteria as it currently stands. Therefore, we invite you to submit a revised version of the manuscript that addresses the points raised during the review process.

==============================

Please ensure that the second reviewer comments are addressed properly.

==============================

Please submit your revised manuscript by Apr 03 2026 11:59PM. If you will need more time than this to complete your revisions, please reply to this message or contact the journal office at plosone@plos.org. When you're ready to submit your revision, log on to https://www.editorialmanager.com/pone/ and select the 'Submissions Needing Revision' folder to locate your manuscript file.

Please include the following items when submitting your revised manuscript:

- A letter that responds to each point raised by the academic editor and reviewer(s). You should upload this letter as a separate file labeled 'Response to Reviewers'.
- A marked-up copy of your manuscript that highlights changes made to the original version. You should upload this as a separate file labeled 'Revised Manuscript with Track Changes'.
- An unmarked version of your revised paper without tracked changes. You should upload this as a separate file labeled 'Manuscript'.

If you would like to make changes to your financial disclosure, please include your updated statement in your cover letter. Guidelines for resubmitting your figure files are available below the reviewer comments at the end of this letter.

If applicable, we recommend that you deposit your laboratory protocols in protocols.io to enhance the reproducibility of your results. Protocols.io assigns your protocol its own identifier (DOI) so that it can be cited independently in the future. For instructions see: https://journals.plos.org/plosone/s/submission-guidelines#loc-laboratory-protocols. Additionally, PLOS ONE offers an option for publishing peer-reviewed Lab Protocol articles, which describe protocols hosted on protocols.io. Read more information on sharing protocols at https://plos.org/protocols?utm_medium=editorial-email&utm_source=authorletters&utm_campaign=protocols.

We look forward to receiving your revised manuscript.

Kind regards,

Wejdan Shahin

Academic Editor

PLOS One

Journal Requirements:

If the reviewer comments include a recommendation to cite specific previously published works, please review and evaluate these publications to determine whether they are relevant and should be cited. There is no requirement to cite these works unless the editor has indicated otherwise.

[Note: HTML markup is below. Please do not edit.]

Reviewers' comments:

Reviewer's Responses to Questions

**Comments to the Author**

1. If the authors have adequately addressed your comments raised in a previous round of review and you feel that this manuscript is now acceptable for publication, you may indicate that here to bypass the “Comments to the Author” section, enter your conflict of interest statement in the “Confidential to Editor” section, and submit your "Accept" recommendation.

Reviewer #1: All comments have been addressed

Reviewer #2: (No Response)

**********

2. Is the manuscript technically sound, and do the data support the conclusions?

The manuscript must describe a technically sound piece of scientific research with data that supports the conclusions. Experiments must have been conducted rigorously, with appropriate controls, replication, and sample sizes. The conclusions must be drawn appropriately based on the data presented.

Reviewer #1: Yes

Reviewer #2: Yes

**********

3. Has the statistical analysis been performed appropriately and rigorously?

Reviewer #1: Yes

Reviewer #2: Yes

**********

4. Have the authors made all data underlying the findings in their manuscript fully available?

The PLOS Data policy requires authors to make all data underlying the findings described in their manuscript fully available without restriction, with rare exception (please refer to the Data Availability Statement in the manuscript PDF file). The data should be provided as part of the manuscript or its supporting information, or deposited to a public repository. For example, in addition to summary statistics, the data points behind means, medians and variance measures should be available. If there are restrictions on publicly sharing data—e.g. participant privacy or use of data from a third party—those must be specified.

Reviewer #1: Yes

Reviewer #2: Yes

**********

5. Is the manuscript presented in an intelligible fashion and written in standard English?

PLOS ONE does not copyedit accepted manuscripts, so the language in submitted articles must be clear, correct, and unambiguous. Any typographical or grammatical errors should be corrected at revision, so please note any specific errors here.

Reviewer #1: Yes

Reviewer #2: Yes

**********

6. Review Comments to the Author

Please use the space provided to explain your answers to the questions above. You may also include additional comments for the author, including concerns about dual publication, research ethics, or publication ethics. (Please upload your review as an attachment if it exceeds 20,000 characters)

Reviewer #1: Dear Academic Editor and Authors,

Thank you for the opportunity to review this manuscript, “Profiles of medication literacy and associated factors among young and middle-aged hypertensive patients in China: a latent profile analysis.”The study is a multicenter cross-sectional survey of 544 hypertensive adults aged 18–59 years recruited from two tertiary hospitals (July 15, 2024–November 15, 2024) and applies latent profile analysis (LPA) to identify medication-literacy subgroups, followed by logistic regression to examine associated factors.

Below I put forward 3 points to modify the proposal, for your reference�

1.Data Availability statement appears internally inconsistent; this must be corrected to meet PLOS expectations for reproducibility.

In the submission materials, “No—some restrictions will apply” is selected, yet the accompanying statement indicates that all data are contained within the manuscript/Supporting Information.

2.Methods are not yet sufficiently reproducible for LPA + regression (inputs, missing data, and classification uncertainty).

The manuscript indicates use of SPSS 26.0 and Mplus 8.3 and lists fit indices (AIC/BIC/aBIC, entropy, LMR, BLRT), but key implementation details are still missing (e.g., which variables were entered into LPA—18 items vs. domain scores; whether variables were standardized; random starts; missing-data handling).

3.Interpretation should be more cautious given cross-sectional design and potential information bias from assisted completion.

The study is cross-sectional, sampled from tertiary hospitals, and a subset of respondents had questionnaires read aloud and completed by researchers, which may introduce measurement or social-desirability bias.

The study addresses an important applied question and the LPA-derived profiles are promising.However, I recommend making the above 3 changes, primarily to (i) resolve the Data Availability inconsistency, (ii) strengthen reproducibility and appropriately handle classification uncertainty, and (iii) tighten causal language and bias/generalizability discussion in line with the design.

Reviewer #2: Reviewer Comments

Introduction

The introduction addresses an important topic; however, it would benefit from stronger conceptual grounding. Key variables such as self-efficacy and illness perception are discussed, yet they are not clearly situated within an established theoretical framework, which somewhat limits the scientific coherence of the study.

In addition, the rationale for adopting a latent profile approach is introduced rather abruptly, with limited discussion of the shortcomings of prior variable-centered analyses. A more explicit justification of this methodological choice would strengthen the argument.

Finally, the decision to focus on young and middle-aged adults (18–59 years) requires clearer justification, particularly in terms of why this specific age group warrants distinct investigation.

Methods

While the methodological structure is generally appropriate, several aspects require clarification. The manuscript does not provide a formal power analysis or detailed justification for sample adequacy in the context of latent profile analysis, where class stability and model complexity are important considerations.

Furthermore, essential modeling details related to the latent profile procedure—such as estimation methods, model assumptions, and safeguards against local maxima—are not sufficiently described, which limits reproducibility.

The regression strategy also requires clearer explanation. The criteria for variable selection and control of potential confounders are not fully articulated, and there is no indication that multicollinearity among predictors was assessed.

Finally, the recruitment of participants from only two tertiary hospitals in a single city raises concerns regarding external validity and potential selection bias.

Results

The selection of the three-profile solution would benefit from a more comprehensive justification, particularly given that fit indices continued to improve with additional classes. A clearer discussion of the trade-offs underlying this decision is warranted.

In addition, latent class membership was used in subsequent regression analyses without addressing Potential classification uncertainty may influence the accuracy of parameter estimates.

The results section places strong emphasis on statistical significance, while offering limited discussion of effect sizes and their clinical implications.

Overall, the presentation remains largely descriptive, with limited analytical depth and no robustness or sensitivity analyses to support the stability of the identified profiles.

Discussion

The discussion summarizes the findings effectively but would benefit from deeper analytical engagement. Much of the section reiterates results rather than exploring underlying mechanisms or situating the findings within broader theoretical perspectives.

Given the cross-sectional design, some interpretations and practice-oriented recommendations should be framed more cautiously to avoid implying causal relationships. A more explicit reflection on methodological limitations, particularly those related to the latent profile approach, would also strengthen this section.

Limitations and Conclusion

Although general limitations are acknowledged, there is limited discussion of issues specific to the modeling approach, such as model selection uncertainty or classification error. Moreover, the connection between stated limitations and the strength of the conclusions could be more clearly articulated.

The conclusion largely restates the main findings without clearly highlighting the study’s theoretical or methodological contribution. Practical recommendations appear somewhat stronger than what can be supported by an observational design and would benefit from more cautious framing.

Abstract

The abstract would benefit from a more balanced structure. Clinical implications appear somewhat overstated given the study design, and key limitations are not mentioned. Additionally, methodological details receive relatively greater emphasis than interpretive insights, which could be better prioritized in a concise summary.

**********

7. PLOS authors have the option to publish the peer review history of their article (what does this mean?). If published, this will include your full peer review and any attached files.

If you choose “no”, your identity will remain anonymous but your review may still be made public.

**Do you want your identity to be public for this peer review?**  For information about this choice, including consent withdrawal, please see our Privacy Policy.

Reviewer #1: No

Reviewer #2: No

**********

[NOTE: If reviewer comments were submitted as an attachment file, they will be attached to this email and accessible via the submission site. Please log into your account, locate the manuscript record, and check for the action link "View Attachments". If this link does not appear, there are no attachment files.]

To ensure your figures meet our technical requirements, please review our figure guidelines: https://journals.plos.org/plosone/s/figures

You may also use PLOS’s free figure tool, NAAS, to help you prepare publication quality figures: https://journals.plos.org/plosone/s/figures#loc-tools-for-figure-preparation.

NAAS will assess whether your figures meet our technical requirements by comparing each figure against our figure specifications.

# Author comment: Author response to Decision Letter 2

**DOI:** 10.1371/journal.pone.0352857.r004

1 Apr 2026

Dear Reviewers,

We appreciate the opportunity to revise our manuscript titled “Profiles of medication literacy and associated factors among young and middle-aged hypertensive patients in China: a latent profile analysis (PONE-D-25-50007R1)” and are grateful for the insightful comments provided by the reviewers. Those comments are all valuable and very helpful for revising and improving our paper, as well as the important guiding significance to our researches. In the following, we have provided detailed responses to each of the reviewers’ comments. Revised portion are marked in blue in the revised manuscript with track changes paper. We have tried our best to make all the revisions clear, and we hope that the revised manuscript meets the requirements for publication.

Attachment Submitted filename: Response_to_reviewers_auresp_2.doc

# Peer review: Decision Letter 2

**DOI:** 10.1371/journal.pone.0352857.r005

**Authors:** Wejdan Shahin, Wejdan Shahin, Ilse Bloom

16 Apr 2026

PONE-D-25-50007R2

Profiles of medication literacy and associated factors among young and middle-aged hypertensive patients in China: a latent profile analysis

PLOS One

Dear Dr. wang,

Thank you for submitting your manuscript to PLOS ONE. After careful consideration, we feel that it has merit but does not fully meet PLOS ONE’s publication criteria as it currently stands. Therefore, we invite you to submit a revised version of the manuscript that addresses the points raised during the review process.

The manuscript has been further evaluated by two reviewers, and their comments are available below.

Could you please carefully revise the manuscript to address all comments raised?

Please submit your revised manuscript by May 31 2026 11:59PM. If you will need more time than this to complete your revisions, please reply to this message or contact the journal office at plosone@plos.org. When you're ready to submit your revision, log on to https://www.editorialmanager.com/pone/ and select the 'Submissions Needing Revision' folder to locate your manuscript file.

Please include the following items when submitting your revised manuscript:

- A letter that responds to each point raised by the academic editor and reviewer(s). You should upload this letter as a separate file labeled 'Response to Reviewers'.
- A marked-up copy of your manuscript that highlights changes made to the original version. You should upload this as a separate file labeled 'Revised Manuscript with Track Changes'.
- An unmarked version of your revised paper without tracked changes. You should upload this as a separate file labeled 'Manuscript'.

If you would like to make changes to your financial disclosure, please include your updated statement in your cover letter. Guidelines for resubmitting your figure files are available below the reviewer comments at the end of this letter.

If applicable, we recommend that you deposit your laboratory protocols in protocols.io to enhance the reproducibility of your results. Protocols.io assigns your protocol its own identifier (DOI) so that it can be cited independently in the future. For instructions see: https://journals.plos.org/plosone/s/submission-guidelines#loc-laboratory-protocols. Additionally, PLOS ONE offers an option for publishing peer-reviewed Lab Protocol articles, which describe protocols hosted on protocols.io. Read more information on sharing protocols at https://plos.org/protocols?utm_medium=editorial-email&utm_source=authorletters&utm_campaign=protocols.

As the corresponding author, your ORCID iD is verified in the submission system and will appear in the published article. PLOS supports the use of ORCID, and we encourage all coauthors to register for an ORCID iD and use it as well. Please encourage your coauthors to verify their ORCID iD within the submission system before final acceptance, as unverified ORCID iDs will not appear in the published article. *Only*  the individual author can complete the verification step; PLOS staff *cannot*  verify ORCID iDs on behalf of authors.

We look forward to receiving your revised manuscript.

Kind regards,

Ilse Bloom

Staff Editor

PLOS One

Journal Requirements:

If the reviewer comments include a recommendation to cite specific previously published works, please review and evaluate these publications to determine whether they are relevant and should be cited. There is no requirement to cite these works unless the editor has indicated otherwise.

[Note: HTML markup is below. Please do not edit.]

Reviewers' comments:

Reviewer's Responses to Questions

**Comments to the Author**

1. If the authors have adequately addressed your comments raised in a previous round of review and you feel that this manuscript is now acceptable for publication, you may indicate that here to bypass the “Comments to the Author” section, enter your conflict of interest statement in the “Confidential to Editor” section, and submit your "Accept" recommendation.

Reviewer #1: All comments have been addressed

Reviewer #2: All comments have been addressed

**********

2. Is the manuscript technically sound, and do the data support the conclusions?

The manuscript must describe a technically sound piece of scientific research with data that supports the conclusions. Experiments must have been conducted rigorously, with appropriate controls, replication, and sample sizes. The conclusions must be drawn appropriately based on the data presented.

Reviewer #1: Yes

Reviewer #2: Yes

**********

3. Has the statistical analysis been performed appropriately and rigorously?

Reviewer #1: Yes

Reviewer #2: Yes

**********

4. Have the authors made all data underlying the findings in their manuscript fully available?

The PLOS Data policy requires authors to make all data underlying the findings described in their manuscript fully available without restriction, with rare exception (please refer to the Data Availability Statement in the manuscript PDF file). The data should be provided as part of the manuscript or its supporting information, or deposited to a public repository. For example, in addition to summary statistics, the data points behind means, medians and variance measures should be available. If there are restrictions on publicly sharing data—e.g. participant privacy or use of data from a third party—those must be specified.

Reviewer #1: Yes

Reviewer #2: Yes

**********

5. Is the manuscript presented in an intelligible fashion and written in standard English?

PLOS ONE does not copyedit accepted manuscripts, so the language in submitted articles must be clear, correct, and unambiguous. Any typographical or grammatical errors should be corrected at revision, so please note any specific errors here.

Reviewer #1: Yes

Reviewer #2: Yes

**********

6. Review Comments to the Author

Please use the space provided to explain your answers to the questions above. You may also include additional comments for the author, including concerns about dual publication, research ethics, or publication ethics. (Please upload your review as an attachment if it exceeds 20,000 characters)

Reviewer #1: The study revolves around the heterogeneity of drug literacy in young and middle-aged patients with hypertension, adopts a cross-sectional design, consecutively enrolls 544 patients from two tertiary hospitals, and conducts a potential profile analysis using the four dimensions of C-MLSHP-R, and analyzes the relationship between drug literacy and risk factors, and further test the related factors with multinomial Logistic regression. The authors identified three subtypes of drug literacy: low, moderate, and high, and reported that education level, family income, social support, self-efficacy, and disease perception were significantly associated with subtype attribution.

According to PLOS ONE's criteria of“Only judging scientific rigor”, the advantages of this paper are clear research questions, complete ethical approval and informed consent, and more complete modeling details than common cross-sectional studies, the random starting point, VIF and split-half sensitivity analysis are supplemented, which shows that the authors have made efforts to improve the reproducibility and stability of the results.

However, there are still some key issues that affect the judgment of publication:

1� data availability statements are still not enough to satiPLOSPOne ONE's reproducibility requirements.Data availability information is still unclear from the current manuscript package, and the authors themselves admit to previous inconsistencies during the round-trip review.

Recommendation: Please include additional subjects in flow charts or tables and at least report:

(a) specific decision rules for 29 invalid questionnaires;

(b) the proportion missing for each core variable/scale;

(C) LPA versus treatment of missingness used in regression;

(d) in the absence of missing data, state explicitly“No missing data for variables entered into LPA/regression”.

2)The external validity is limited, while the practical recommendations are still slightly strong.

The sample was collected from two tertiary hospitals in the same city in China, and a small number of illiterate patients were filled in by the offspring, which may introduce selection bias and information bias. Although the authors mention this in their limitations, the conclusion“Provides a theoretical basis for the development of intervention programs for caregivers” and“Should be individualized,” which is slightly forward in the hierarchy of evidence.

3)On the basis of univariate significant variables and theoretically relevant confounders, multinomial Logistic regression was entered; Table 4 also encodes variables such as age, place of residence, occupational status, type of work, and blood pressure monitoring. However, Table 5 ultimately reported only five categories of variables: education, income, PSSS, GSES, and BIPQ, complete adjustment for age, place of residence, occupation, type of work, blood pressure monitoring, or theoretical confounders such as sex was not seen.

4)Language and grammar errors are still more, it is recommended to Polish professional English.

5)There are spelling and typographical errors in the table. table1 and table 5.

6)It is suggested that the scale reliability index of the current sample should be supplemented in the text

7)References and format details should be unified.

8)In the results section, it is suggested to reduce“Statistically significant” repetitive statements and increase model interpretation

Reviewer #2: The manuscript has improved and most comments have been addressed well.

Only one minor point:

Please briefly clarify the sample size justification for the latent profile analysis in the Methods section.

Overall, the manuscript is suitable for publication after this minor revision.

**********

7. PLOS authors have the option to publish the peer review history of their article (what does this mean?). If published, this will include your full peer review and any attached files.

If you choose “no”, your identity will remain anonymous but your review may still be made public.

**Do you want your identity to be public for this peer review?**  For information about this choice, including consent withdrawal, please see our Privacy Policy.

Reviewer #1: No

Reviewer #2: **Yes:**  Abdulelah Al-Ganad

**********

[NOTE: If reviewer comments were submitted as an attachment file, they will be attached to this email and accessible via the submission site. Please log into your account, locate the manuscript record, and check for the action link "View Attachments". If this link does not appear, there are no attachment files.]

To ensure your figures meet our technical requirements, please review our figure guidelines: https://journals.plos.org/plosone/s/figures

You may also use PLOS’s free figure tool, NAAS, to help you prepare publication quality figures: https://journals.plos.org/plosone/s/figures#loc-tools-for-figure-preparation.

NAAS will assess whether your figures meet our technical requirements by comparing each figure against our figure specifications.

# Author comment: Author response to Decision Letter 3

**DOI:** 10.1371/journal.pone.0352857.r006

18 May 2026

Dear the academic editor and Reviewers,

We appreciate the opportunity to revise our manuscript titled “Profiles of medication literacy and associated factors among young and middle-aged hypertensive patients in China: a latent profile analysis (PONE-D-25-50007R2)” and are grateful for the insightful comments provided by the reviewers. Those comments are all valuable and very helpful for revising and improving our paper, as well as the important guiding significance to our researches. In the following, we have provided detailed responses to each of the reviewers’ comments. Revised portion are marked in blue in the revised manuscript with track changes paper. We have tried our best to make all the revisions clear, and we hope that the revised manuscript meets the requirements for publication. The main corrections in the paper and the responses to the reviewer’s comments are as following:

Responses to the reviewer’s comments:

Reviewer #1: The study revolves around the heterogeneity of drug literacy in young and middle-aged patients with hypertension, adopts a cross-sectional design, consecutively enrolls 544 patients from two tertiary hospitals, and conducts a potential profile analysis using the four dimensions of C-MLSHP-R, and analyzes the relationship between drug literacy and risk factors, and further test the related factors with multinomial Logistic regression. The authors identified three subtypes of drug literacy: low, moderate, and high, and reported that education level, family income, social support, self-efficacy, and disease perception were significantly associated with subtype attribution. According to PLOS ONE’s criteria of “Only judging scientific rigor”, the advantages of this paper are clear research questions, complete ethical approval and informed consent, and more complete modeling details than common cross-sectional studies, the random starting point, VIF and split-half sensitivity analysis are supplemented, which shows that the authors have made efforts to improve the reproducibility and stability of the results. However, there are still some key issues that affect the judgment of publication:

1. Data availability statements are still not enough to sati PLOSONE’s reproducibility requirements. Data availability information is still unclear from the current manuscript package, and the authors themselves admit to previous inconsistencies during the round-trip review.

Recommendation: Please include additional subjects in flow charts or tables and at least report: (a) specific decision rules for 29 invalid questionnaires; (b) the proportion missing for each core variable/scale; (C) LPA versus treatment of missingness used in regression; (d) in the absence of missing data, state explicitly “No missing data for variables entered into LPA/regression”.

Response: We would like to express our sincere gratitude to the reviewer for your insightful comments and valuable feedback. We have supplemented additional subjects in results portion in this text.

2. The external validity is limited, while the practical recommendations are still slightly strong. The sample was collected from two tertiary hospitals in the same city in China, and a small number of illiterate patients were filled in by the offspring, which may introduce selection bias and information bias. Although the authors mention this in their limitations, the conclusion “Provides a theoretical basis for the development of intervention programs for caregivers” and “Should be individualized,” which is slightly forward in the hierarchy of evidence.

Response: We would like to express our sincere gratitude to the reviewer for your insightful comments and valuable feedback. We have corrected the language in the conclusion, which is slightly forward in the hierarchy of evidence.

3. On the basis of univariate significant variables and theoretically relevant confounders, multinomial Logistic regression was entered; Table 4 also encodes variables such as age, place of residence, occupational status, type of work, and blood pressure monitoring. However, Table 5 ultimately reported only five categories of variables: education, income, PSSS, GSES, and BIPQ, complete adjustment for age, place of residence, occupation, type of work, blood pressure monitoring, or theoretical confounders such as sex was not seen.

Response: Thank you for this important and constructive comment. We agree that the covariates included in the multinomial logistic regression model should be reported more transparently. In the revised manuscript, we re-ran the multinomial logistic regression using a fully adjusted model. In addition to education level, per capita monthly household income, PSSS, GSES, and BIPQ, we included theoretically relevant sociodemographic and disease-management covariates, including age, sex, place of residence, occupational/work-related status, and blood pressure monitoring.

During model re-specification, we found that occupational status and type of work were highly overlapping in the dataset, particularly because retired participants were simultaneously coded as “retired/neither” for type of work. To avoid multicollinearity and unstable parameter estimates, we handled these two variables as occupational/work-related status in the adjusted model. All retained covariates were kept in the model regardless of statistical significance. We have revised the Statistical analysis section to clarify the model-building strategy, added sex/gender coding to the variable assignment table, and expanded Table 5 to present the fully adjusted multinomial logistic regression results, including both statistically significant and non-significant covariates. The Results section and table footnotes have also been revised accordingly.

4. Language and grammar errors are still more, it is recommended to Polish professional English.

Response: We would like to express our sincere gratitude to the reviewer for your insightful comments and valuable feedback. We have tried our best to improve the language and grammar errors in this manuscript and made some changes in the manuscript.

5. There are spelling and typographical errors in the table. table1 and table 5.

Response: We would like to express our sincere gratitude to the reviewer for your insightful comments and valuable feedback. We have checked and corrected the spelling and typographical errors in the table1 and table 5.

6. It is suggested that the scale reliability index of the current sample should be supplemented in the text.

Response: We would like to express our sincere gratitude to the reviewer for your insightful comments and valuable feedback. The reliability of the scales was assessed using Cronbach’s α coefficient in this study. We have supplemented in the text.

7. References and format details should be unified.

Response: We would like to express our sincere gratitude to the reviewer for your insightful comments and valuable feedback. We have checked and corrected references and format details in the text.

8. In the results section, it is suggested to reduce“Statistically significant” repetitive statements and increase model interpretation.

Response: We would like to express our sincere gratitude to the reviewer for your insightful comments and valuable feedback. In the results section, we have reduced the “Statistically significant” repetitive statements.

Reviewer #2: The manuscript has improved and most comments have been addressed well. Only one minor point: Please briefly clarify the sample size justification for the latent profile analysis in the Methods section. Overall, the manuscript is suitable for publication after this minor revision.

Response: We would like to express our sincere gratitude to the reviewer for your insightful comments and valuable feedback. We have clarified the sample size justification for the latent profile analysis in the Methods section.

We tried our best to improve the manuscript and made some changes in the manuscript. These changes will not influence the content and framework of the paper. And here we did not list the changes but marked in blue in the revised paper.

We appreciate for Editors/Reviewers’ warm work earnestly, and hope that the correction will meet with approval.

Once again, thank you very much for your comments and suggestions.

Best regards!

Yours sincerely,

Rui Wang

Attachment Submitted filename: Response_to_reviewers_auresp_3.doc

# Peer review: Decision Letter 3

**DOI:** 10.1371/journal.pone.0352857.r007

**Authors:** Wejdan Shahin, Wejdan Shahin, Ilse Bloom, Fan Chen

26 May 2026

PONE-D-25-50007R3

Profiles of medication literacy and associated factors among young and middle-aged hypertensive patients in China: a latent profile analysis

PLOS One

Dear Dr. wang,

Thank you for submitting your manuscript to PLOS ONE. After careful consideration, we feel that it has merit but does not fully meet PLOS ONE’s publication criteria as it currently stands. Therefore, we invite you to submit a revised version of the manuscript that addresses the points raised during the review process.

Please submit your revised manuscript by Jul 10 2026 11:59PM. If you will need more time than this to complete your revisions, please reply to this message or contact the journal office at plosone@plos.org. When you're ready to submit your revision, log on to https://www.editorialmanager.com/pone/ and select the 'Submissions Needing Revision' folder to locate your manuscript file.

Please include the following items when submitting your revised manuscript:

- A letter that responds to each point raised by the academic editor and reviewer(s). You should upload this letter as a separate file labeled 'Response to Reviewers'.
- A marked-up copy of your manuscript that highlights changes made to the original version. You should upload this as a separate file labeled 'Revised Manuscript with Track Changes'.
- An unmarked version of your revised paper without tracked changes. You should upload this as a separate file labeled 'Manuscript'.

If you would like to make changes to your financial disclosure, please include your updated statement in your cover letter. Guidelines for resubmitting your figure files are available below the reviewer comments at the end of this letter.

If applicable, we recommend that you deposit your laboratory protocols in protocols.io to enhance the reproducibility of your results. Protocols.io assigns your protocol its own identifier (DOI) so that it can be cited independently in the future. For instructions see: https://journals.plos.org/plosone/s/submission-guidelines#loc-laboratory-protocols. Additionally, PLOS ONE offers an option for publishing peer-reviewed Lab Protocol articles, which describe protocols hosted on protocols.io. Read more information on sharing protocols at https://plos.org/protocols?utm_medium=editorial-email&utm_source=authorletters&utm_campaign=protocols.

As the corresponding author, your ORCID iD is verified in the submission system and will appear in the published article. PLOS supports the use of ORCID, and we encourage all coauthors to register for an ORCID iD and use it as well. Please encourage your coauthors to verify their ORCID iD within the submission system before final acceptance, as unverified ORCID iDs will not appear in the published article. *Only*  the individual author can complete the verification step; PLOS staff *cannot*  verify ORCID iDs on behalf of authors.

We look forward to receiving your revised manuscript.

Kind regards,

Fan Chen

Academic Editor

PLOS One

Journal Requirements:

If the reviewer comments include a recommendation to cite specific previously published works, please review and evaluate these publications to determine whether they are relevant and should be cited. There is no requirement to cite these works unless the editor has indicated otherwise.

Please review your reference list to ensure that it is complete and correct. If you have cited papers that have been retracted, please include the rationale for doing so in the manuscript text, or remove these references and replace them with relevant current references. Any changes to the reference list should be mentioned in the rebuttal letter that accompanies your revised manuscript. If you need to cite a retracted article, indicate the article’s retracted status in the References list and also include a citation and full reference for the retraction notice.

Additional Editor Comments:

While the manuscript has undergone proper revision, please carefully revise it further following the expert review comments.

[Note: HTML markup is below. Please do not edit.]

Reviewers' comments:

Reviewer's Responses to Questions

**Comments to the Author**

1. If the authors have adequately addressed your comments raised in a previous round of review and you feel that this manuscript is now acceptable for publication, you may indicate that here to bypass the “Comments to the Author” section, enter your conflict of interest statement in the “Confidential to Editor” section, and submit your "Accept" recommendation.

Reviewer #1: All comments have been addressed

Reviewer #2: All comments have been addressed

**********

2. Is the manuscript technically sound, and do the data support the conclusions?

The manuscript must describe a technically sound piece of scientific research with data that supports the conclusions. Experiments must have been conducted rigorously, with appropriate controls, replication, and sample sizes. The conclusions must be drawn appropriately based on the data presented.

Reviewer #1: Yes

Reviewer #2: Yes

**********

3. Has the statistical analysis been performed appropriately and rigorously?

Reviewer #1: Yes

Reviewer #2: Yes

**********

4. Have the authors made all data underlying the findings in their manuscript fully available?

The PLOS Data policy requires authors to make all data underlying the findings described in their manuscript fully available without restriction, with rare exception (please refer to the Data Availability Statement in the manuscript PDF file). The data should be provided as part of the manuscript or its supporting information, or deposited to a public repository. For example, in addition to summary statistics, the data points behind means, medians and variance measures should be available. If there are restrictions on publicly sharing data—e.g. participant privacy or use of data from a third party—those must be specified.

Reviewer #1: Yes

Reviewer #2: No

**********

5. Is the manuscript presented in an intelligible fashion and written in standard English?

PLOS ONE does not copyedit accepted manuscripts, so the language in submitted articles must be clear, correct, and unambiguous. Any typographical or grammatical errors should be corrected at revision, so please note any specific errors here.

Reviewer #1: Yes

Reviewer #2: Yes

**********

6. Review Comments to the Author

Please use the space provided to explain your answers to the questions above. You may also include additional comments for the author, including concerns about dual publication, research ethics, or publication ethics. (Please upload your review as an attachment if it exceeds 20,000 characters)

Reviewer #1: 1.Use “moderate medication literacy” consistently instead of alternating between “medium” and “moderate.”

Replace full-width symbols such as “�” with standard mathematical symbols.

2.Use χ² or “Chi-square” instead of “X2.”

3.Explain the scoring range and direction for each scale and each C-MLSHP-R domain.

4.Please check all DOI formatting; at least one DOI appears to contain an extra space.

Avoid speculative statements in the Discussion unless clearly marked as possible explanations. For example, “Maybe people with high incomes…” should be rewritten in formal academic language.

5.The funding statement should include whether the funder had any role in study design, data collection and analysis, decision to publish, or manuscript preparation.

6.Please avoid overstating external validity. Since the sample was recruited from two tertiary hospitals in one city, the study should be described as a two-hospital, single-city, hospital-based cross-sectional study.

Overall, the manuscript has a clear research question and uses an appropriate person-centered method. The three-profile solution appears plausible based on the reported fit indices and sensitivity analysis. Nevertheless, the unresolved data availability issue, unstable multinomial regression results, inconsistencies in model reporting, and remaining language problems prevent acceptance in the present form. I recommend minor revision.

Reviewer #2: Reviewer’s comments

Thank you for the authors’ efforts in revising the manuscript. The paper has clearly improved, and most of the previous comments have been addressed. I only have a few minor suggestions that may help improve the clarity and transparency of the manuscript before final acceptance.

1-Sample size justification for LPA

The sample size is mentioned as adequate, but it would be helpful if the authors briefly clarify why 544 participants are sufficient for latent profile analysis.

2-Data availability

Please provide a clearer Data Availability Statement, including the repository name, DOI, accession number, or link. If the data cannot be shared openly, the reason should be briefly explained.

3-Invalid questionnaires and missing data

The explanation of the 29 excluded questionnaires is clear, but it may be better placed in the Methods section or shown in a simple participant flow chart.

4-Table 5 and unstable ORs

Some odds ratios in Table 5 appear unstable because of zero cases in certain categories. The authors may either address this statistically, for example by merging sparse categories or using penalized multinomial logistic regression, or clearly mention that these estimates should be interpreted with caution.

5-Discussion and practical recommendations

The intervention-related recommendations are useful, but they should be expressed more cautiously. Please clarify that these recommendations are preliminary and hypothesis-generating, rather than clinical guidelines.

6-Generalizability

Please clarify that although the sample size may be adequate for LPA, the recruitment from two hospitals in one city may limit the generalizability of the findings.

Recommendation:

Accept after minor revisions.

**********

7. PLOS authors have the option to publish the peer review history of their article (what does this mean?). If published, this will include your full peer review and any attached files.

If you choose “no”, your identity will remain anonymous but your review may still be made public.

**Do you want your identity to be public for this peer review?**  For information about this choice, including consent withdrawal, please see our Privacy Policy.

Reviewer #1: No

Reviewer #2: **Yes:**  Abdulelah Al-Ganad

**********

[NOTE: If reviewer comments were submitted as an attachment file, they will be attached to this email and accessible via the submission site. Please log into your account, locate the manuscript record, and check for the action link "View Attachments". If this link does not appear, there are no attachment files.]

To ensure your figures meet our technical requirements, please review our figure guidelines: https://journals.plos.org/plosone/s/figures

You may also use PLOS’s free figure tool, NAAS, to help you prepare publication quality figures: https://journals.plos.org/plosone/s/figures#loc-tools-for-figure-preparation.

NAAS will assess whether your figures meet our technical requirements by comparing each figure against our figure specifications.

# Author comment: Author response to Decision Letter 4

**DOI:** 10.1371/journal.pone.0352857.r008

2 Jun 2026

Responses to the reviewer’s comments:

Reviewer #1:

1. Use “moderate medication literacy” consistently instead of alternating between “medium” and “moderate.” Replace full-width symbols such as “�” with standard mathematical symbols.

Response: We would like to express our sincere gratitude to the reviewer for your insightful comments and valuable feedback. We have corrected these imperfection in the revised manuscript with track changes.

2.Use χ² or “Chi-square” instead of “X2.”

Response: We would like to express our sincere gratitude to the reviewer for your insightful comments and valuable feedback. We have also corrected these imperfection in the revised manuscript with track changes.

3.Explain the scoring range and direction for each scale and each C-MLSHP-R domain.

Response: We would like to express our sincere gratitude to the reviewer for your insightful comments and valuable feedback. We have supplemented the scoring range and direction for each scale and each C-MLSHP-R domain in the revised manuscript with track changes.

4. Please check all DOI formatting; at least one DOI appears to contain an extra space. Avoid speculative statements in the Discussion unless clearly marked as possible explanations. For example, “Maybe people with high incomes…” should be rewritten in formal academic language.

Response: We would like to express our sincere gratitude to the reviewer for your insightful comments and valuable feedback. We have also corrected DOI formatting and speculative statements in the revised manuscript with track changes.

5. The funding statement should include whether the funder had any role in study design, data collection and analysis, decision to publish, or manuscript preparation.

Response: We would like to express our sincere gratitude to the reviewer for your insightful comments and valuable feedback. We have supplemented the funding statement in the title page in the revised manuscript with track changes.

6. Please avoid overstating external validity. Since the sample was recruited from two tertiary hospitals in one city, the study should be described as a two-hospital, single-city, hospital-based cross-sectional study.

Response: We would like to express our sincere gratitude to the reviewer for your insightful comments and valuable feedback. We have also corrected these imperfection in the revised manuscript with track changes.

Reviewer #2: Thank you for the authors’ efforts in revising the manuscript. The paper has clearly improved, and most of the previous comments have been addressed. I only have a few minor suggestions that may help improve the clarity and transparency of the manuscript before final acceptance.

1. Sample size justification for LPA

The sample size is mentioned as adequate, but it would be helpful if the authors briefly clarify why 544 participants are sufficient for latent profile analysis.

Response: We would like to express our sincere gratitude to the reviewer for your insightful comments and valuable feedback. Actually, in our paper, the references (14, 19 and 20) are all shown that “best-practice and review results concluded that a minimum sample size of about 500 should lead to enough accuracy in identifying a correct number of latent profiles”.

2. Data availability

Please provide a clearer Data Availability Statement, including the repository name, DOI, accession number, or link. If the data cannot be shared openly, the reason should be briefly explained.

Response: We would like to express our sincere gratitude to the reviewer for your insightful comments and valuable feedback. We have supplemented the data availability statement in the title page in the revised manuscript with track changes.

3. Invalid questionnaires and missing data

The explanation of the 29 excluded questionnaires is clear, but it may be better placed in the Methods section or shown in a simple participant flow chart.

Response: We would like to express our sincere gratitude to the reviewer for your insightful comments and valuable feedback. The explanation of the 29 excluded questionnaires have placed in the Methods section in the revised manuscript with track changes.

4. Table 5 and unstable ORs

Some odds ratios in Table 5 appear unstable because of zero cases in certain categories. The authors may either address this statistically, for example by merging sparse categories or using penalized multinomial logistic regression, or clearly mention that these estimates should be interpreted with caution.

Response: We would like to express our sincere gratitude to the reviewer for your insightful comments and valuable feedback. As the reviewer noted that some OR values in Table 5 exhibit wide confidence intervals. This phenomenon is attributable to data sparsity in the high medication literacy profile (Profile 3, n = 79). This sparse data situation leads to quasi-complete separation in logistic regression, where the maximum likelihood estimator becomes unstable, producing inflated OR values and extremely wide confidence intervals. We acknowledge this limitation and have taken the following steps in the revised manuscript:

a) Added cautionary interpretation in Results: It should be noted that some OR estimates (e.g., education: junior middle school and below) exhibited wide confidence intervals due to sparse cell counts in the high medication literacy profile (n = 79). These estimates should be interpreted with caution, and the direction of association rather than the precise magnitude is more informative in these cases."

b) Added acknowledgment in Limitations: “Fourth, the relatively small sample size of the high medication literacy profile (n = 79) limited the number of covariates that could be reliably included in the multinomial logistic regression model. The sparse data in this subgroup led to wide confidence intervals for some OR estimates, particularly for education and income categories. Future studies with larger sample sizes are needed to provide more precise estimates and to allow for comprehensive covariate adjustment including demographic confounders such as age, sex, and place of residence”.

5. Discussion and practical recommendations

The intervention-related recommendations are useful, but they should be expressed more cautiously. Please clarify that these recommendations are preliminary and hypothesis-generating, rather than clinical guidelines.

Response: We would like to express our sincere gratitude to the reviewer for your insightful comments and valuable feedback. We have supplemented the statement in the revised manuscript with track changes.

6. Generalizability

Please clarify that although the sample size may be adequate for LPA, the recruitment from two hospitals in one city may limit the generalizability of the findings.

Response: We would like to express our sincere gratitude to the reviewer for your insightful comments and valuable feedback. We have supplemented the statement in the manuscript.

Once again, thank you very much for your comments and suggestions.

Best regards!

Yours sincerely,

Rui Wang

Attachment Submitted filename: Response_to_reviewers_auresp_4.doc

# Peer review: Decision Letter 4

**DOI:** 10.1371/journal.pone.0352857.r009

**Authors:** Wejdan Shahin, Wejdan Shahin, Ilse Bloom, Fan Chen, Fan Chen

16 Jun 2026

Profiles of medication literacy and associated factors among young and middle-aged hypertensive patients in China: a latent profile analysis

PONE-D-25-50007R4

Dear Dr. Wang,

We’re pleased to inform you that your manuscript has been judged scientifically suitable for publication and will be formally accepted for publication once it meets all outstanding technical requirements.

Within one week, you’ll receive an e-mail detailing the required amendments. When these have been addressed, you’ll receive a formal acceptance letter and your manuscript will be scheduled for publication.

An invoice will be generated when your article is formally accepted. Please note, if your institution has a publishing partnership with PLOS and your article meets the relevant criteria, all or part of your publication costs will be covered. Please make sure your user information is up-to-date by logging into Editorial Manager at Editorial Manager® and clicking the ‘Update My Information' link at the top of the page. For questions related to billing, please contact billing support.

If your institution or institutions have a press office, please notify them about your upcoming paper to help maximize its impact. If they’ll be preparing press materials, please inform our press team as soon as possible -- no later than 48 hours after receiving the formal acceptance. Your manuscript will remain under strict press embargo until 2 pm Eastern Time on the date of publication. For more information, please contact onepress@plos.org.

Kind regards,

Fan Chen

Academic Editor

PLOS One

Additional Editor Comments (optional):

The author has made satisfactory revisions based on the reviewers' comments. The manuscript is recommended for acceptance.

Reviewers' comments:

Reviewer's Responses to Questions

**Comments to the Author**

1. If the authors have adequately addressed your comments raised in a previous round of review and you feel that this manuscript is now acceptable for publication, you may indicate that here to bypass the “Comments to the Author” section, enter your conflict of interest statement in the “Confidential to Editor” section, and submit your "Accept" recommendation.

Reviewer #1: All comments have been addressed

Reviewer #2: All comments have been addressed

**********

2. Is the manuscript technically sound, and do the data support the conclusions?

The manuscript must describe a technically sound piece of scientific research with data that supports the conclusions. Experiments must have been conducted rigorously, with appropriate controls, replication, and sample sizes. The conclusions must be drawn appropriately based on the data presented.

Reviewer #1: Yes

Reviewer #2: Yes

**********

3. Has the statistical analysis been performed appropriately and rigorously?

Reviewer #1: Yes

Reviewer #2: Yes

**********

4. Have the authors made all data underlying the findings in their manuscript fully available?

The PLOS Data policy requires authors to make all data underlying the findings described in their manuscript fully available without restriction, with rare exception (please refer to the Data Availability Statement in the manuscript PDF file). The data should be provided as part of the manuscript or its supporting information, or deposited to a public repository. For example, in addition to summary statistics, the data points behind means, medians and variance measures should be available. If there are restrictions on publicly sharing data—e.g. participant privacy or use of data from a third party—those must be specified.

Reviewer #1: Yes

Reviewer #2: Yes

**********

5. Is the manuscript presented in an intelligible fashion and written in standard English?

PLOS ONE does not copyedit accepted manuscripts, so the language in submitted articles must be clear, correct, and unambiguous. Any typographical or grammatical errors should be corrected at revision, so please note any specific errors here.

Reviewer #1: Yes

Reviewer #2: Yes

**********

6. Review Comments to the Author

Please use the space provided to explain your answers to the questions above. You may also include additional comments for the author, including concerns about dual publication, research ethics, or publication ethics. (Please upload your review as an attachment if it exceeds 20,000 characters)

Reviewer #1: Thank you for the opportunity to review the revised manuscript. The manuscript has improved compared with the previous version, and several concerns have been addressed. However, I think some important issues still need to be resolved before the manuscript can be considered suitable for publication in PLOS ONE.

The research question is clear and appropriate for a cross-sectional observational study. The manuscript focuses on identifying medication literacy profiles among young and middle-aged patients with hypertension and examining associated factors.

The use of latent profile analysis is generally suitable for the person-centered objective of the study. The authors report key model fit indices, including AIC, BIC, aBIC, entropy, LMR, and BLRT, and provide a rationale for selecting the three-profile solution.

The revised manuscript has improved in transparency. The authors added scoring ranges for the scales, reported internal consistency coefficients, clarified the exclusion of invalid questionnaires, added a data availability statement, and acknowledged limitations related to cross-sectional design and limited generalizability.

Reviewer #2: I appreciate the authors’ efforts in revising the manuscript. Overall, I feel that they have responded well to the reviewers’ comments and have improved the manuscript clearly.

I have no major remaining concerns. The manuscript only needs minor language polishing and some Word/formatting corrections before final acceptance.

Recommendation: Accept after minor language and formatting corrections.

**********

7. PLOS authors have the option to publish the peer review history of their article (what does this mean?). If published, this will include your full peer review and any attached files.

If you choose “no”, your identity will remain anonymous but your review may still be made public.

**Do you want your identity to be public for this peer review?**  For information about this choice, including consent withdrawal, please see our Privacy Policy.

Reviewer #1: No

Reviewer #2: **Yes:**  Abdulelah Al-Ganad

**********

# Editor report: Acceptance letter

**DOI:** 10.1371/journal.pone.0352857.r010

**Authors:** Wejdan Shahin, Wejdan Shahin, Ilse Bloom, Fan Chen, Fan Chen

PONE-D-25-50007R4

PLOS One

Dear Dr. Wang,

I'm pleased to inform you that your manuscript has been deemed suitable for publication in PLOS One. Congratulations! Your manuscript is now being handed over to our production team.

At this stage, our production department will prepare your paper for publication. This includes ensuring the following:

* All references, tables, and figures are properly cited

* All relevant supporting information is included in the manuscript submission,

* There are no issues that prevent the paper from being properly typeset

You will receive further instructions from the production team, including instructions on how to review your proof when it is ready. Please keep in mind that we are working through a large volume of accepted articles, so please give us a few days to review your paper and let you know the next and final steps.

Lastly, if your institution or institutions have a press office, please let them know about your upcoming paper now to help maximize its impact. If they'll be preparing press materials, please inform our press team within the next 48 hours. Your manuscript will remain under strict press embargo until 2 pm Eastern Time on the date of publication. For more information, please contact onepress@plos.org.

You will receive an invoice from PLOS for your publication fee after your manuscript has reached the completed accept phase. If you receive an email requesting payment before acceptance or for any other service, this may be a phishing scheme. Learn how to identify phishing emails and protect your accounts at https://explore.plos.org/phishing.

If we can help with anything else, please email us at customercare@plos.org.

Thank you for submitting your work to PLOS ONE and supporting open access.

Kind regards,

PLOS ONE Editorial Office Staff

on behalf of

Dr. Fan Chen

Academic Editor

PLOS One
